# Supplementary material for: Molecular characterization and antibiotic resistance of Acinetobacter baumannii in cerebrospinal fluid and blood
Source: PLoS One. 2021 Feb 22;16(2):e0247418. doi: 10.1371/journal.pone.0247418 (PMC7899338; doi:10.1371/journal.pone.0247418)
Supplement: S1 Table — T/C: ticarcillin/clavulanic acid; P/T: piperacillin/tazobactam; CAZ: ceftazidime; CSL: cefoperazone/sulbactam; FEP: cefepime; IPM: imipenem; MEM: meropenem; TOB: tobramycin; CIP: ciprofloxacin; LEV: levofloxacin; MNO: minocycline; TGC: tigecycline; COL: colistin; SXT: sulfamethoxazole/trimethoprim. (DOCX) [file pone.0247418.s001.docx]

S1 Table Minimum Inhibitory Concentrations (MICs) of the tested antimicrobial agents

T/C: ticarcillin/clavulanic acid; P/T: piperacillin/tazobactam; CAZ: ceftazidime; CSL: cefoperazone/sulbactam; FEP: cefepime; IPM: imipenem; MEM: meropenem; TOB: tobramycin; CIP: ciprofloxacin; LEV: levofloxacin; MNO: minocycline; TGC: tigecycline; COL: colistin; SXT: sulfamethoxazole/trimethoprim.

| **strain** | **T/C** | **P/T** | **CAZ** | **CSL** | **FEP** | **IPM** | **MEM** | **TOB** | **CIP** | **LEV** | **MNO** | **TGC** | **COL** | **SXT** |
| --- | --- | --- | --- | --- | --- | --- | --- | --- | --- | --- | --- | --- | --- | --- |
| AB01 | ≥128 | ≥128 | ≥64 | 32 | 16 | ≥16 | ≥16 | ≥16 | ≥4 | ≥8 | 8 | 2 | ≤0.5 | ≥320 |
| AB02 | ≥128 | ≥128 | ≥64 | ≥64 | ≥32 | ≥16 | ≥16 | ≥16 | ≥4 | 4 | 4 | 1 | ≤0.5 | ≥320 |
| AB03 | ≥128 | ≥128 | ≥64 | ≥64 | ≥32 | 8 | 8 | ≥16 | ≥4 | 4 | 4 | 1 | ≤0.5 | ≥320 |
| AB04 | ≥128 | ≥128 | ≥64 | ≥64 | ≥32 | ≥16 | ≥16 | ≥16 | ≥4 | 4 | 4 | 1 | ≤0.5 | ≥320 |
| AB05 | ≥128 | ≥128 | ≥64 | 32 | 16 | ≥16 | 8 | ≥16 | ≥4 | ≥8 | ≤1 | 2 | ≤0.5 | ≥320 |
| AB06 | ≥128 | ≥128 | ≥64 | 32 | 16 | ≥16 | 8 | ≥16 | ≥4 | ≥8 | ≤1 | 1 | ≤0.5 | ≥320 |
| AB07 | ≥128 | ≥128 | ≥64 | ≤8 | ≥32 | ≥16 | ≥16 | ≥16 | ≥4 | ≥8 | ≥16 | ≥8 | ≤0.5 | ≥320 |
| AB08 | ≥128 | ≥128 | ≥64 | 32 | ≥32 | ≥16 | ≥16 | ≥16 | ≥4 | ≥8 | 8 | 2 | ≤0.5 | ≤20 |
| AB09 | ≥128 | ≥128 | ≥64 | 16 | 16 | ≥16 | ≥16 | ≥16 | ≥4 | ≥8 | 4 | 2 | ≤0.5 | ≤20 |
| AB10 | ≥128 | ≥128 | ≥64 | 32 | 16 | ≥16 | ≥16 | ≥16 | ≥4 | ≥8 | 4 | 2 | ≤0.5 | 160 |
| AB11 | ≥128 | ≥128 | ≥64 | 32 | 16 | ≥16 | ≥16 | ≥16 | ≥4 | ≥8 | 4 | 2 | ≤0.5 | 160 |
| AB12 | ≥128 | ≥128 | ≥64 | ≤8 | 16 | 8 | 8 | ≥16 | ≥4 | ≥8 | 8 | 4 | ≤0.5 | 40 |
| AB13 | ≥128 | ≥128 | ≥64 | 32 | 16 | 1 | 0.5 | ≥16 | ≥4 | 4 | 4 | 1 | ≤0.5 | 160 |
| AB14 | ≥128 | ≥128 | ≥64 | ≥64 | 16 | ≥16 | ≥16 | ≥16 | ≥4 | ≥8 | 8 | 4 | ≤0.5 | ≥320 |
| AB15 | ≥128 | ≥128 | ≥64 | 16 | 16 | ≥16 | ≥16 | ≥16 | ≥4 | ≥8 | ≥16 | 4 | ≤0.5 | ≥320 |
| AB16 | ≥128 | ≥128 | ≥64 | 16 | 16 | ≥16 | ≥16 | ≥16 | ≥4 | ≥8 | ≥16 | ≥8 | ≤0.5 | ≥320 |
| AB17 | ≥128 | ≥128 | ≥64 | 32 | 16 | ≥16 | ≥16 | ≥16 | ≥4 | 4 | 4 | 2 | ≤0.5 | 160 |
| AB18 | ≤8 | ≤4 | 2 | ≤8 | 1 | ≤0.25 | ≤0.25 | ≤1 | ≤0.25 | ≤0.12 | ≤1 | ≤0.5 | ≤0.5 | ≤20 |
| AB19 | ≥128 | ≥128 | ≥64 | 32 | ≥32 | ≥16 | ≥16 | ≥16 | ≥4 | ≥8 | 2 | 1 | ≤0.5 | ≥320 |
| AB20 | ≥128 | ≥128 | ≥64 | 32 | ≥32 | ≥16 | ≥16 | ≥16 | ≥4 | ≥8 | ≤1 | 1 | ≤0.5 | ≥320 |
| AB21 | ≥128 | ≥128 | ≥64 | 32 | ≥32 | ≥16 | ≥16 | ≥16 | ≥4 | ≥8 | 4 | 1 | ≤0.5 | ≤20 |
| AB22 | ≥128 | ≥128 | ≥64 | 32 | ≥32 | ≥16 | ≥16 | ≥16 | ≥4 | ≥8 | 2 | 2 | ≤0.5 | ≥320 |
| AB23 | ≥128 | ≥128 | ≥64 | 32 | ≥32 | ≥16 | ≥16 | ≥16 | ≥4 | ≥8 | 4 | 1 | ≤0.5 | ≥320 |
| AB24 | ≥128 | ≥128 | ≥64 | ≥64 | ≥32 | ≥16 | ≥16 | ≥16 | ≥4 | 4 | 2 | 1 | ≤0.5 | ≥320 |
| AB25 | ≥128 | ≥128 | ≥64 | ≥64 | 16 | ≥16 | ≥16 | 1 | ≥4 | ≥8 | 4 | 2 | ≤0.5 | ≤20 |
| AB26 | ≥128 | ≥128 | ≥64 | 32 | ≥32 | ≥16 | ≥16 | ≥16 | ≥4 | 4 | ≤1 | ≤0.5 | ≤0.5 | ≥320 |
| AB27 | ≥128 | ≥128 | ≥64 | ≥64 | ≥32 | ≥16 | ≥16 | 4 | ≥4 | ≥8 | 8 | 2 | ≤0.5 | ≤20 |
| AB28 | ≤8 | ≤4 | 2 | ≤8 | 2 | ≤0.25 | ≤0.25 | ≤1 | ≤0.25 | ≤0.12 | ≤1 | ≤0.5 | ≤0.5 | ≤20 |
| AB29 | 64 | 16 | 2 | ≤8 | 8 | 2 | ≥16 | ≤1 | ≤0.25 | ≤0.12 | ≤1 | ≤0.5 | ≤0.5 | ≤20 |
| AB30 | ≥128 | ≥128 | ≥64 | ≥64 | ≥32 | ≥16 | ≥16 | ≥16 | ≥4 | ≥8 | 8 | ≥8 | ≤0.5 | ≤20 |
| AB31 | ≥128 | ≥128 | ≥64 | ≥64 | 16 | ≥16 | ≥16 | ≥16 | ≥4 | ≥8 | 8 | 1 | ≤0.5 | ≥320 |
| AB32 | ≥128 | ≥128 | ≥64 | 32 | ≥32 | ≥16 | ≥16 | ≥16 | ≥4 | ≥8 | 4 | 1 | ≤0.5 | ≤20 |
| AB33 | ≥128 | ≥128 | ≥64 | 16 | ≥32 | ≥16 | ≥16 | ≥16 | ≥4 | 4 | 4 | ≤0.5 | ≤0.5 | ≥320 |
| AB34 | ≥128 | ≥128 | ≥64 | ≥64 | ≥32 | ≥16 | ≥16 | ≥16 | ≥4 | 8 | ≤1 | 1 | ≤0.5 | 160 |
| AB35 | ≥128 | ≥128 | ≥64 | ≥64 | ≥32 | ≥16 | ≥16 | ≥16 | ≥4 | 8 | 4 | 1 | ≤0.5 | ≥320 |
| AB36 | ≥128 | ≥128 | ≥64 | 32 | ≥32 | ≥16 | ≥16 | ≥16 | ≥4 | 8 | 4 | 1 | ≤0.5 | ≤20 |
| AB37 | ≥128 | ≥128 | ≥64 | ≥64 | 16 | ≥16 | ≥16 | ≥16 | ≥4 | ≥8 | ≥16 | 4 | ≤0.5 | ≥320 |
| AB38 | ≥128 | ≥128 | ≥64 | 32 | 16 | ≥16 | ≥16 | ≥16 | ≥4 | ≥8 | 4 | 2 | ≤0.5 | ≤20 |
| AB39 | ≥128 | ≥128 | ≥64 | ≥64 | ≥32 | ≥16 | ≥16 | ≤1 | ≥4 | ≥8 | ≤1 | 1 | ≤0.5 | ≤20 |
| AB40 | ≥128 | ≥128 | ≥64 | 32 | ≥32 | ≥16 | ≥16 | ≥16 | ≥4 | ≥8 | 4 | 1 | ≤0.5 | ≥320 |
| AB41 | ≥128 | ≥128 | ≥64 | 16 | 16 | ≥16 | ≥16 | ≥16 | ≥4 | 4 | 4 | 1 | ≤0.5 | ≥320 |
| AB42 | ≥128 | ≥128 | ≥64 | ≥64 | 16 | ≥16 | ≥16 | ≤1 | ≥4 | ≥8 | ≥16 | 4 | ≤0.5 | ≤20 |
| AB43 | ≥128 | ≥128 | ≥64 | ≥64 | ≥32 | ≥16 | ≥16 | ≥16 | ≥4 | ≥8 | 4 | 4 | ≤0.5 | ≥320 |
| AB44 | ≥128 | ≥128 | ≥64 | 32 | 16 | ≥16 | ≥16 | ≥16 | ≥4 | ≥8 | ≤1 | 2 | ≤0.5 | ≥320 |
| AB45 | ≥128 | ≥128 | ≥64 | ≥64 | ≥32 | ≥16 | ≥16 | ≤1 | ≥4 | ≥8 | 4 | 2 | ≤0.5 | ≥320 |
| AB46 | ≥128 | ≥128 | ≥64 | ≥64 | ≥32 | ≥16 | ≥16 | ≥16 | ≥4 | ≥8 | ≥16 | 4 | ≤0.5 | ≥320 |
| AB47 | ≥128 | ≥128 | ≥64 | 32 | ≥32 | ≥16 | ≥16 | ≥16 | ≥4 | ≥8 | 4 | 2 | ≤0.5 | ≤20 |
| AB48 | ≥128 | ≥128 | ≥64 | 32 | ≥32 | ≥16 | ≥16 | ≤1 | ≥4 | ≥8 | 8 | 2 | ≤0.5 | ≥320 |
| AB49 | ≥128 | ≥128 | ≥64 | ≥64 | ≥32 | ≥16 | ≥16 | ≥16 | ≥4 | ≥8 | 1 | 1 | ≤0.5 | 160 |
| AB50 | ≥128 | ≥128 | ≥64 | ≥64 | ≥32 | ≥16 | ≥16 | ≥16 | ≥4 | ≥8 | 8 | 2 | ≤0.5 | ≥320 |
| AB51 | ≥128 | ≥128 | ≥64 | 16 | ≥32 | ≥16 | ≥16 | ≥16 | ≥4 | ≥8 | 4 | ≤0.5 | ≤0.5 | ≤20 |
| AB52 | ≥128 | ≥128 | ≥64 | ≥64 | ≥32 | ≥16 | ≥16 | ≥16 | ≥4 | 4 | 2 | 1 | ≤0.5 | ≤20 |
| AB53 | ≥128 | ≥128 | ≥64 | 32 | 16 | ≥16 | ≥16 | ≥16 | ≥4 | ≥8 | ≥16 | 4 | ≤0.5 | ≥320 |
| AB54 | ≥128 | ≥128 | ≥64 | 16 | 16 | ≥16 | ≥16 | ≥16 | ≥4 | ≥8 | 8 | 1 | ≤0.5 | ≥320 |
| AB55 | ≥128 | ≥128 | ≥64 | 16 | 16 | ≥16 | ≥16 | ≥16 | ≥4 | ≥8 | 4 | 2 | ≤0.5 | ≤20 |
| AB56 | ≤8 | ≤4 | 2 | ≤8 | 2 | ≤0.25 | ≤0.25 | ≤1 | ≤0.25 | ≤0.12 | ≤1 | ≤0.5 | ≤0.5 | ≤20 |
| AB57 | ≥128 | ≥128 | ≥64 | ≥64 | ≥32 | ≥16 | ≥16 | ≤1 | ≥4 | 4 | 4 | 1 | ≤0.5 | ≥320 |
| AB58 | ≥128 | ≥128 | ≥64 | ≥64 | 16 | ≥16 | ≥16 | ≤1 | ≥4 | ≥8 | ≥16 | 4 | ≤0.5 | ≤20 |
| AB59 | ≥128 | ≥128 | ≥64 | 32 | ≥32 | ≥16 | ≥16 | ≥16 | ≥4 | ≥8 | 8 | ≥8 | ≤0.5 | ≤20 |
| AB60 | ≥128 | ≥128 | ≥64 | 32 | ≥32 | ≥16 | ≥16 | ≥16 | ≥4 | ≥8 | 4 | 1 | ≤0.5 | ≥320 |
| AB61 | ≥128 | ≥128 | ≥64 | 16 | 16 | ≥16 | ≥16 | ≥16 | ≥4 | ≥8 | ≥16 | 2 | ≤0.5 | ≥320 |
| AB62 | ≥128 | ≥128 | ≥64 | ≥64 | ≥32 | ≥16 | ≥16 | ≥16 | ≥4 | 4 | 2 | 1 | ≤0.5 | ≤20 |
| AB63 | ≥128 | ≥128 | ≥64 | ≥64 | ≥32 | ≥16 | ≥16 | ≥16 | ≥4 | ≥8 | 8 | 2 | ≤0.5 | 160 |
| AB64 | ≥128 | ≥128 | ≥64 | ≥64 | ≥32 | ≥16 | ≥16 | 4 | ≥4 | ≥8 | 4 | 1 | ≤0.5 | ≤20 |
| AB65 | ≥128 | ≥128 | ≥64 | ≥64 | ≥32 | ≥16 | ≥16 | ≥1 | ≥4 | ≥8 | ≥16 | 2 | ≤0.5 | ≤20 |
| AB66 | ≥128 | ≥128 | ≥64 | ≥64 | 16 | ≥16 | ≥16 | ≥16 | ≥4 | 4 | 4 | ≤0.5 | ≤0.5 | ≤20 |
| AB67 | ≥128 | ≥128 | ≥64 | 32 | 16 | ≥16 | ≥16 | ≥16 | ≥4 | ≥8 | 4 | 2 | ≤0.5 | 160 |
| AB68 | ≥128 | ≥128 | ≥64 | 32 | 16 | ≥16 | ≥16 | ≥16 | ≥4 | ≥8 | ≥16 | ≥8 | ≤0.5 | ≥320 |
| AB69 | ≥128 | ≥128 | ≥64 | 16 | ≥32 | ≥16 | ≥16 | ≥16 | ≥4 | ≥8 | ≥16 | 2 | ≤0.5 | ≤20 |
| AB70 | 64 | 16 | 2 | ≤8 | 8 | 2 | ≥16 | ≤1 | ≤0.25 | ≤0.12 | ≤1 | ≤0.5 | ≤0.5 | ≤20 |
| AB71 | ≥128 | ≥128 | ≥64 | 32 | 16 | ≥16 | ≥16 | ≥16 | ≥4 | 4 | 8 | 1 | ≤0.5 | ≥320 |
| AB72 | ≥128 | ≥128 | ≥64 | ≥64 | ≥32 | ≥16 | ≥16 | ≥16 | ≥4 | ≥8 | ≥16 | ≥8 | ≤0.5 | ≤20 |
| AB73 | ≥128 | ≥128 | ≥64 | 32 | 16 | ≥16 | ≥16 | 4 | ≥4 | ≥8 | ≥16 | 4 | ≤0.5 | ≤20 |
| AB74 | ≥128 | ≥128 | ≥64 | ≥64 | ≥32 | ≥16 | ≥16 | ≥16 | ≥4 | ≥8 | 8 | 2 | ≤0.5 | ≥320 |
| AB75 | ≥128 | ≥128 | ≥64 | ≥64 | 16 | ≥16 | ≥16 | ≥16 | ≥4 | ≥8 | ≥16 | 2 | ≤0.5 | ≥320 |
| AB76 | ≥128 | ≥128 | ≥64 | 32 | ≥32 | ≥16 | ≥16 | ≥16 | ≥4 | ≥8 | 2 | 1 | ≤0.5 | ≤20 |
| AB77 | ≥128 | ≥128 | ≥64 | ≥64 | 16 | ≥16 | ≥16 | ≥16 | ≥4 | ≥8 | ≤1 | 1 | ≤0.5 | ≥320 |
| AB78 | ≥128 | ≥128 | ≥64 | 32 | 16 | ≥16 | ≥16 | ≥16 | ≥4 | ≥8 | ≥16 | ≥8 | ≤0.5 | ≥320 |
| AB79 | ≥128 | ≥128 | ≥64 | ≥64 | ≥32 | ≥16 | ≥16 | ≥16 | ≥4 | ≥8 | ≥16 | 4 | ≤0.5 | ≥320 |
| AB80 | ≤8 | ≤4 | 2 | ≤8 | 0.5 | ≤0.25 | ≤0.25 | ≤1 | ≤0.25 | ≤0.12 | ≤1 | ≤0.5 | ≤0.5 | ≤20 |
| AB81 | ≥128 | ≥128 | ≥64 | ≥64 | ≥32 | ≥16 | ≥16 | ≥16 | ≥4 | 4 | 4 | 1 | ≤0.5 | ≥320 |
| AB82 | ≥128 | ≥128 | ≥64 | ≥64 | ≥32 | ≥16 | ≥16 | ≥16 | ≥4 | ≥8 | 8 | 2 | ≤0.5 | ≤20 |
| AB83 | ≤8 | ≤4 | 2 | ≤8 | 2 | ≤0.25 | ≤0.25 | ≤1 | ≤0.25 | ≤0.12 | ≤1 | ≤0.5 | ≤0.5 | ≤20 |
| AB84 | ≥128 | ≥128 | ≥64 | ≥64 | 16 | ≥16 | ≥16 | ≥16 | ≥4 | ≥8 | ≥16 | 4 | ≤0.5 | 160 |
| AB85 | ≥128 | ≥128 | ≥64 | 32 | ≥32 | ≥16 | ≥16 | ≥16 | ≥4 | ≥8 | 8 | 2 | ≤0.5 | ≤20 |
| AB86 | ≥128 | ≥128 | ≥64 | ≥64 | ≥32 | ≥16 | ≥16 | ≥16 | ≥4 | ≥8 | 8 | 4 | ≤0.5 | ≥320 |
| AB87 | ≥128 | ≥128 | ≥64 | ≥64 | 16 | ≥16 | ≥16 | ≥16 | ≥4 | ≥8 | 8 | 2 | ≤0.5 | 160 |
| AB88 | ≥128 | ≥128 | ≥64 | 32 | ≥32 | ≥16 | ≥16 | ≥16 | ≥4 | ≥8 | 4 | 1 | ≤0.5 | ≤20 |
| AB89 | ≥128 | ≥128 | ≥64 | ≥64 | 16 | ≥16 | ≥16 | ≤1 | ≥4 | ≥8 | ≤1 | 1 | ≤0.5 | 160 |
| AB90 | ≥128 | ≥128 | ≥64 | ≥64 | ≥32 | ≥16 | ≥16 | ≥16 | ≥4 | ≥8 | 8 | 1 | ≤0.5 | ≤20 |
| AB91 | ≥128 | ≥128 | ≥64 | ≥64 | 16 | ≥16 | ≥16 | ≤1 | ≥4 | ≥8 | ≤1 | 1 | ≤0.5 | 160 |
| AB92 | ≥128 | ≥128 | ≥64 | ≥64 | 16 | ≥16 | ≥16 | ≤1 | ≥4 | 4 | 4 | ≤0.5 | ≤0.5 | ≤20 |
| AB93 | ≥128 | ≥128 | ≥64 | ≥64 | 16 | ≥16 | ≥16 | ≥16 | ≥4 | ≥8 | 8 | 2 | ≤0.5 | ≥320 |
| AB94 | ≥128 | ≥128 | ≥64 | ≥64 | ≥32 | ≥16 | ≥16 | ≥16 | ≥4 | ≥8 | 8 | 1 | ≤0.5 | ≥320 |
