# Supplementary material for: Molecular characterization and antibiotic resistance of Acinetobacter baumannii in cerebrospinal fluid and blood
Source: PLoS One. 2021 Feb 22;16(2):e0247418. doi: 10.1371/journal.pone.0247418 (PMC7899338; doi:10.1371/journal.pone.0247418)
Supplement: S2 Table — (DOCX) [file pone.0247418.s002.docx]

S2 Table Allelic profiles of the *A. baumannii* strains used in this study

| **Strain** | ***gltA*** | ***gyrB*** | ***gdhB*** | ***recA*** | ***cpn60*** | ***gpi*** | ***rpoD*** | **ST** |
| --- | --- | --- | --- | --- | --- | --- | --- | --- |
| AB01 | 1 | 3 | 3 | 2 | 2 | 97 | 3 | 208 |
| AB02 | 1 | 3 | 3 | 2 | 2 | 96 | 3 | 195 |
| AB03 | 1 | 3 | 3 | 2 | 2 | 96 | 3 | 195 |
| AB04 | 1 | 3 | 3 | 2 | 2 | 96 | 3 | 195 |
| AB05 | 1 | 81 | 3 | 2 | 2 | 16 | 3 | 381 |
| AB06 | 1 | 81 | 3 | 2 | 2 | 16 | 3 | 381 |
| AB07 | 1 | 64 | 3 | 2 | 2 | 106 | 3 | 1779 |
| AB08 | 1 | 3 | 3 | 2 | 2 | 96 | 3 | 195 |
| AB09 | 1 | 3 | 3 | 2 | 2 | 96 | 3 | 195 |
| AB10 | 1 | 3 | 3 | 2 | 2 | 160 | 3 | 540 |
| AB11 | 1 | 3 | 3 | 2 | 2 | 160 | 3 | 540 |
| AB12 | 1 | 3 | 3 | 2 | 2 | 160 | 3 | 540 |
| AB13 | 1 | 3 | 3 | 2 | 2 | 97 | 3 | 208 |
| AB14 | 1 | 34 | 3 | 2 | 2 | 178 | 3 | 1967(new ST) |
| AB15 | 1 | 3 | 3 | 2 | 2 | 113 | 3 | 1968(new ST) |
| AB16 | 1 | 3 | 3 | 2 | 2 | 113 | 3 | 1968(new ST) |
| AB17 | 1 | 3 | 3 | 2 | 2 | 160 | 3 | 540 |
| AB18 | 1 | 12 | 56 | 1 | 1 | 61 | 45 | 377 |
| AB19 | 1 | 34 | 3 | 2 | 2 | 178 | 3 | 1967(new ST) |
| AB20 | 1 | 3 | 3 | 2 | 2 | 96 | 3 | 195 |
| AB21 | 1 | 34 | 3 | 2 | 2 | 178 | 3 | 1967(new ST) |
| AB22 | 1 | 34 | 3 | 2 | 2 | 178 | 3 | 1967(new ST) |
| AB23 | 1 | 3 | 3 | 2 | 2 | 96 | 3 | 195 |
| AB24 | 1 | 12 | 3 | 2 | 2 | 103 | 3 | 469 |
| AB25 | 1 | 3 | 3 | 2 | 2 | 160 | 3 | 540 |
| AB26 | 1 | 15 | 2 | 28 | 1 | 107 | 32 | 229 |
| AB27 | 1 | 3 | 3 | 2 | 2 | 96 | 3 | 195 |
| AB28 | 1 | 12 | 56 | 1 | 1 | 61 | 45 | 377 |
| AB29 | 1 | 17 | 135 | 12 | 23 | 98 | 6 | 1969(new ST) |
| AB30 | 1 | 3 | 3 | 2 | 2 | 96 | 3 | 195 |
| AB31 | 1 | 3 | 3 | 2 | 2 | 97 | 3 | 208 |
| AB32 | 1 | 3 | 3 | 2 | 2 | 94 | 3 | 191 |
| AB33 | 1 | 64 | 3 | 2 | 2 | 106 | 3 | 1779 |
| AB34 | 1 | 3 | 3 | 2 | 2 | 94 | 3 | 191 |
| AB35 | 1 | 3 | 3 | 2 | 2 | 97 | 3 | 208 |
| AB36 | 1 | 3 | 3 | 2 | 2 | 94 | 3 | 191 |
| AB37 | 1 | 3 | 3 | 2 | 2 | 106 | 3 | 369 |
| AB38 | 1 | 3 | 3 | 2 | 2 | 160 | 3 | 540 |
| AB39 | 1 | 81 | 3 | 2 | 2 | 16 | 3 | 381 |
| AB40 | 1 | 3 | 3 | 2 | 2 | 94 | 3 | 191 |
| AB41 | 1 | 64 | 3 | 2 | 2 | 106 | 3 | 1779 |
| AB42 | 1 | 3 | 3 | 2 | 2 | 201 | 3 | 800 |
| AB43 | 1 | 3 | 3 | 2 | 2 | 96 | 3 | 195 |
| AB44 | 1 | 81 | 3 | 2 | 2 | 16 | 3 | 381 |
| AB45 | 1 | 3 | 3 | 2 | 2 | 97 | 3 | 208 |
| AB46 | 1 | 3 | 3 | 2 | 2 | 96 | 3 | 195 |
| AB47 | 1 | 3 | 3 | 2 | 2 | 96 | 3 | 195 |
| AB48 | 1 | 3 | 3 | 2 | 2 | 160 | 3 | 540 |
| AB49 | 1 | 3 | 3 | 2 | 2 | 94 | 3 | 191 |
| AB50 | 1 | 3 | 3 | 2 | 2 | 96 | 3 | 195 |
| AB51 | 1 | 3 | 3 | 2 | 2 | 96 | 3 | 195 |
| AB52 | 1 | 3 | 3 | 2 | 2 | 96 | 3 | 195 |
| AB53 | 1 | 3 | 3 | 2 | 2 | 113 | 3 | 1968(new ST) |
| AB54 | 1 | 3 | 3 | 2 | 2 | 97 | 3 | 208 |
| AB55 | 1 | 3 | 3 | 2 | 2 | 96 | 3 | 195 |
| AB56 | 1 | 12 | 12 | 11 | 4 | 103 | 3 | 373 |
| AB57 | 1 | 3 | 3 | 2 | 2 | 142 | 3 | 451 |
| AB58 | 1 | 3 | 3 | 2 | 2 | 106 | 3 | 369 |
| AB59 | 1 | 3 | 3 | 2 | 2 | 96 | 3 | 195 |
| AB60 | 1 | 3 | 3 | 2 | 2 | 96 | 3 | 195 |
| AB61 | 1 | 3 | 3 | 2 | 2 | 97 | 3 | 208 |
| AB62 | 1 | 3 | 3 | 2 | 2 | 96 | 3 | 195 |
| AB63 | 1 | 3 | 3 | 2 | 2 | 160 | 4 | 1970(new ST) |
| AB64 | 1 | 3 | 3 | 2 | 2 | 96 | 3 | 195 |
| AB65 | 1 | 3 | 3 | 2 | 2 | 97 | 3 | 208 |
| AB66 | 1 | 3 | 3 | 2 | 2 | 96 | 3 | 195 |
| AB67 | 1 | 3 | 3 | 2 | 2 | 160 | 3 | 540 |
| AB68 | 1 | 3 | 3 | 2 | 2 | 113 | 3 | 1968(new ST) |
| AB69 | 1 | 3 | 3 | 2 | 2 | 96 | 3 | 195 |
| AB70 | 1 | 17 | 135 | 12 | 23 | 98 | 6 | 1969(new ST) |
| AB71 | 1 | 3 | 3 | 2 | 2 | 160 | 3 | 540 |
| AB72 | 1 | 3 | 3 | 2 | 2 | 106 | 3 | 369 |
| AB73 | 1 | 3 | 3 | 2 | 2 | 113 | 3 | 1968(new ST) |
| AB74 | 1 | 3 | 3 | 2 | 2 | 97 | 3 | 208 |
| AB75 | 1 | 3 | 3 | 2 | 2 | 106 | 3 | 369 |
| AB76 | 1 | 3 | 3 | 2 | 2 | 94 | 3 | 191 |
| AB77 | 1 | 3 | 3 | 2 | 2 | 94 | 3 | 191 |
| AB78 | 1 | 3 | 3 | 2 | 2 | 113 | 3 | 1968(new ST) |
| AB79 | 1 | 3 | 3 | 2 | 2 | 97 | 3 | 208 |
| AB80 | 1 | 35 | 12 | 11 | 4 | 109 | 3 | 712 |
| AB81 | 1 | 3 | 3 | 2 | 2 | 16 | 3 | 136 |
| AB82 | 1 | 15 | 3 | 2 | 2 | 153 | 3 | 457 |
| AB83 | 36 | 34 | 59 | 28 | 4 | 279 | 3 | 1971(new ST) |
| AB84 | 1 | 3 | 3 | 2 | 2 | 160 | 3 | 540 |
| AB85 | 1 | 3 | 3 | 2 | 2 | 96 | 3 | 195 |
| AB86 | 1 | 3 | 3 | 2 | 2 | 160 | 3 | 540 |
| AB87 | 1 | 3 | 3 | 2 | 2 | 160 | 3 | 540 |
| AB88 | 1 | 3 | 3 | 2 | 2 | 94 | 3 | 191 |
| AB89 | 1 | 81 | 3 | 2 | 2 | 16 | 3 | 381 |
| AB90 | 1 | 3 | 3 | 2 | 2 | 96 | 3 | 195 |
| AB91 | 1 | 81 | 3 | 2 | 2 | 16 | 3 | 381 |
| AB92 | 1 | 3 | 3 | 2 | 2 | 106 | 3 | 369 |
| AB93 | 1 | 3 | 3 | 2 | 2 | 97 | 3 | 208 |
| AB94 | 1 | 3 | 3 | 2 | 2 | 106 | 3 | 369 |
