# Supplementary material for: Molecular characterization and antibiotic resistance of Acinetobacter baumannii in cerebrospinal fluid and blood
Source: PLoS One. 2021 Feb 22;16(2):e0247418. doi: 10.1371/journal.pone.0247418 (PMC7899338; doi:10.1371/journal.pone.0247418)
Supplement: S3 Table — (DOCX) [file pone.0247418.s003.docx]

S3 Table Antibiotics used for the CRAB treatments

| **Strain** | **Antibiotics used for the CRAB treatments** |
| --- | --- |
| AB01 | Cefoperazone/sulbactam, β-lactams |
| AB02 | Tigecycline, Cefoperazone/sulbactam, Levofloxacin |
| AB03 | Tigecycline, Cefoperazone/sulbactam, Levofloxacin |
| AB04 | Tigecycline, Cefoperazone/sulbactam, Levofloxacin |
| AB05 | Tigecycline, Cefoperazone/sulbactam |
| AB06 | Cefoperazone/sulbactam |
| AB07 | Tigecycline, Cefoperazone/sulbactam |
| AB08 | Tigecycline, Biapenem, Levofloxacin |
| AB09 | Tigecycline, Meropenem |
| AB10 | Cefoperazone/sulbactam, Mezlocillin/Sulbactam |
| AB11 | Tigecycline, Cefoperazone/sulbactam, Biapenem, Etimicin |
| AB12 | Tigecycline, Cefoperazone/sulbactam, Minocycline, Etimicin |
| AB14 | Tigecycline, Cefoperazone/sulbactam, Amikacin |
| AB15 | Tigecycline, Cefoperazone/sulbactam, Minocycline, Biapenem, Amikacin |
| AB16 | Tigecycline, Cefoperazone/sulbactam, Imipenem/Cilastatin, Amikacin |
| AB17 | Tigecycline, Cefoperazone/sulbactam, Minocycline, Meropenem, Amikacin, Gentamicin |
| AB19 | Cefoperazone/sulbactam, Meropenem, β-lactams |
| AB20 | Cefoperazone/sulbactam, Minocycline, Meropenem, Gentamicin |
| AB21 | Tigecycline, Cefoperazone/sulbactam, Minocycline, Meropenem, Amikacin, β-lactams |
| AB22 | Cefoperazone/sulbactam, Minocycline, Biapenem |
| AB23 | Tigecycline, Cefoperazone/sulbactam |
| AB24 | Tigecycline, Cefoperazone/sulbactam, Meropenem, Etimicin |
| AB25 | Tigecycline, Cefoperazone/sulbactam, Amikacin, Levofloxacin |
| AB26 | Tigecycline, Minocycline, Mezlocillin/Sulbactam, Amikacin, Levofloxacin, β-lactams |
| AB27 | Tigecycline, Cefoperazone/sulbactam, Amikacin |
| AB29 | Cefoperazone/sulbactam |
| AB30 | Cefoperazone/sulbactam, Etimicin |
| AB31 | Tigecycline, Cefoperazone/sulbactam |
| AB32 | Tigecycline, Cefoperazone/sulbactam, Biapenem, Mezlocillin/Sulbactam, Etimicin |
| AB33 | Tigecycline, Minocycline, Biapenem |
| AB34 | Piperacillin/Tazobactam, Amikacin |
| AB35 | Tigecycline, Cefoperazone/sulbactam, Meropenem, Amikacin |
| AB36 | Tigecycline, Cefoperazone/sulbactam, Minocycline, Amikacin, Levofloxacin, β-lactams |
| AB37 | Tigecycline, Cefoperazone/sulbactam, Meropenem, Levofloxacin |
| AB38 | Tigecycline, Biapenem |
| AB39 | Tigecycline, Meropenem |
| AB40 | Tigecycline, Cefoperazone/sulbactam, colistin, Amikacin, Levofloxacin |
| AB41 | Tigecycline |
| AB42 | Tigecycline, colistin, Etimicin |
| AB43 | Minocycline, β-lactams |
| AB44 | Tigecycline |
| AB45 | Tigecycline, colistin |
| AB46 | Tigecycline, Biapenem |
| AB47 | Cefoperazone/sulbactam, colistin |
| AB48 | Tigecycline, colistin, Piperacillin/Tazobactam, Amikacin |
| AB49 | Tigecycline, Minocycline |
| AB50 | Tigecycline |
| AB51 | Tigecycline, Cefoperazone/sulbactam, Meropenem |
| AB52 | Tigecycline, Minocycline, Meropenem, Piperacillin/Tazobactam |
| AB53 | Tigecycline, Amikacin, Levofloxacin, β-lactams |
| AB54 | Tigecycline |
| AB55 | Tigecycline |
| AB57 | Cefoperazone/sulbactam, Imipenem/Cilastatin, Amikacin |
| AB58 | Cefoperazone/sulbactam, Etimicin, Levofloxacin |
| AB59 | Tigecycline, Cefoperazone/sulbactam, Meropenem, Levofloxacin |
| AB60 | Tigecycline, Cefoperazone/sulbactam, Meropenem |
| AB61 | Tigecycline, Cefoperazone/sulbactam, Imipenem |
| AB62 | Minocycline, Meropenem |
| AB63 | Imipenem/Cilastatin |
| AB64 | Biapenem, Etimicin |
| AB65 | Etimicin, Levofloxacin |
| AB66 | Tigecycline, Biapenem |
| AB67 | Tigecycline, Imipenem/Cilastatin, Amikacin |
| AB68 | Cefoperazone/sulbactam, Biapenem |
| AB69 | Tigecycline, Amikacin |
| AB70 | Tigecycline, Cefoperazone/sulbactam, Imipenem |
| AB71 | Tigecycline, Cefoperazone/sulbactam, Biapenem |
| AB72 | Tigecycline, Imipenem |
| AB73 | Tigecycline, Amikacin |
| AB74 | Tigecycline |
| AB75 | Tigecycline, Cefoperazone/sulbactam |
| AB76 | Biapenem, Amikacin, β-lactams |
| AB77 | Tigecycline, Biapenem |
| AB78 | Cefoperazone/sulbactam |
| AB79 | Tigecycline, Cefoperazone/sulbactam, Meropenem |
| AB81 | Imipenem/Cilastatin |
| AB82 | Tigecycline, Cefoperazone/sulbactam, Biapenem, Levofloxacin, β-lactams |
| AB84 | Tigecycline |
| AB85 | Tigecycline, Mezlocillin/Sulbactam, Levofloxacin |
| AB86 | Tigecycline, Biapenem |
| AB87 | Tigecycline, Meropenem |
| AB88 | Imipenem/Cilastatin |
| AB89 | Etimicin, Piperacillin/Tazobactam |
| AB90 | Tigecycline, colistin |
| AB91 | Piperacillin/Tazobactam, Amikacin |
| AB92 | Cefoperazone/sulbactam, β-lactams |
| AB93 | Cefoperazone/sulbactam, β-lactams |
| AB94 | Tigecycline |
