# Supplementary material for: Molecular characterization and antibiotic resistance of Acinetobacter baumannii in cerebrospinal fluid and blood
Source: PLoS One. 2021 Feb 22;16(2):e0247418. doi: 10.1371/journal.pone.0247418 (PMC7899338; doi:10.1371/journal.pone.0247418)
Supplement: Attachment — Submitted filename: PONE-D-20-30754_reviewer.pdf [file pone.0247418.s004.pdf]

PLOS ONE

Molecular characterization and antibiotic resistance of Acinetobacter baumannii in cerebrospinal fluid and blood

--Manuscript Draft--

**Manuscript Number:**	PONE-D-20-30754
**Article Type:**	Research Article
**Full Title:**	Molecular characterization and antibiotic resistance of Acinetobacter baumannii in cerebrospinal fluid and blood
**Short Title:**	Epidemiology of Acinetobacter baumannii in Cerebrospinal Fluid and Blood
**Corresponding Author:**	Jiazheng Wang<br>The First Affiliated Hospital of Shandong First Medical University<br>Jinan, CHINA
**Keywords:**	Acinetobacter baumannii, cerebrospinal fluid, CSF, blood, blaOXA-23, CC92, carbapenem resistance, infection
**Abstract:**	To identify the antimicrobial resistance, carbapenemases and genetic relatedness of A. baumannii isolates from cerebrospinal fluid (CSF) and blood, a total of 50 nonrepetitive CSF A. baumannii isolates and 44 blood isolates were collected. The resistance phenotypes were determined according to the Clinical and Laboratory Standards Institute guidelines. We performed Polymerase Chain Reaction (PCR) experiments to detect the carbapenem resistance mechanism. Finally, we conducted Multilocus Sequence Typing (MLST) to depict the genetic relatedness of these isolates. We observed that eighty-eight of the 94 isolates collected were resistant to imipenem or meropenem. Among them, the bla OXA-23 gene was the most prevalent carbapenemase gene with a 91.5% (86/94) detection rate, followed by the bla OXA-24 gene that showed a 2.1% (2/94) detection rate isolates. Among all CRAB observations in this study, isolates with the bla OXA-23 gene were resistant to both imipenem and meropenem. However, isolates positive for the bla OXA-24 gene but negative for the bla OXA-23 gene showed an imipenem-sensitive but meropenem-resistant phenotype. The outcome of multilocus sequence typing analysis showed 21 different STs were distinguished, of which ST195 (25.5%), ST540 (12.8%) and ST208 (11.7%) were most frequently observed. Eighty of the 94 isolates (85.1%) were clustered into CC92, and all CC92 isolates showed a carbapenem resistance phenotype (except AB13). Five novel STs were detected, and most of them were CRAB. In conclusion, a high level of carbapenem resistance was detected in this study. The CC92 and bla OXA-23 gene were predominant. Five novel STs were detected, and these new STs require further investigation to understand the nature of and to prevent outbreaks caused by A. baumannii . Our study provides additional observations and epidemiological data of CSF and blood A. baumannii strains, which may improve future infection control measures and aid in potential clinical treatment in hospitals and other clinical settings.
**Order of Authors:**	Xiaohong Shi
	Hong Wang
	Xin Wang
	Huaiqi Jing
	Ran Duan
	Shuai Qin
	Dongyue Lv
	Yufeng Fan
	Zhenzhou Huang
	B. Kyle Stirling
	Lei Zhang

Powered by Editorial Manager® and ProduXion Manager® from Aries Systems Corporation

	Jiazheng Wang
**Additional Information:**	
**Question**	**Response**
**Financial Disclosure**<br><br>Enter a financial disclosure statement that describes the sources of funding for the work included in this submission. Review the submission guidelines for detailed requirements. View published research articles from *PLOS ONE* for specific examples.<br><br>This statement is required for submission and **will appear in the published article** if the submission is accepted. Please make sure it is accurate.<br><br>**Unfunded studies**<br>Enter: *The author(s) received no specific funding for this work.*<br><br>**Funded studies**<br>Enter a statement with the following details:<br>• Initials of the authors who received each award<br>• Grant numbers awarded to each author<br>• The full name of each funder<br>• URL of each funder website<br>• Did the sponsors or funders play any role in the study design, data collection and analysis, decision to publish, or preparation of the manuscript?<br>• **NO** - Include this sentence at the end of your statement: *The funders had no role in study design, data collection and analysis, decision to publish, or preparation of the manuscript.*<br>• **YES** - Specify the role(s) played.<br><br>* typeset	There are no financial conflicts of interest to disclose
**Competing Interests**<br><br>Use the instructions below to enter a competing interest statement for this submission. On behalf of all authors, disclose any competing interests that could be perceived to bias this work—acknowledging all financial support and any other relevant financial or non-financial competing interests.	The authors have declared that no competing interests exist.

Powered by Editorial Manager® and ProduXion Manager® from Aries Systems Corporation

This statement **will appear in the published article** if the submission is accepted. Please make sure it is accurate. View published research articles from *PLOS ONE* for specific examples.<br><br>**NO authors have competing interests**<br><br>Enter: *The authors have declared that no competing interests exist.*<br><br>**Authors with competing interests**<br><br>Enter competing interest details beginning with this statement:<br><br>*I have read the journal's policy and the authors of this manuscript have the following competing interests: [insert competing interests here]*<br><br>* typeset	
**Ethics Statement**<br><br>Enter an ethics statement for this submission. This statement is required if the study involved:<br><br>• Human participants<br>• Human specimens or tissue<br>• Vertebrate animals or cephalopods<br>• Vertebrate embryos or tissues<br>• Field research<br><br>Write "N/A" if the submission does not require an ethics statement.<br><br>General guidance is provided below. Consult the submission guidelines for detailed instructions. **Make sure that all information entered here is included in the Methods section of the manuscript.**	As this study just focus on bacteria alone and does not use any human material or patient information, the Ethics Committee of The First Affiliated Hospital of Shandong First Medical University exempted this study from review and the Review Board also waived the need for informed consent.<br>(We have added this information in the revised manuscript.)

Powered by Editorial Manager® and ProduXion Manager® from Aries Systems Corporation

**Format for specific study types**<br><br>**Human Subject Research (involving human participants and/or tissue)**<br>• Give the name of the institutional review board or ethics committee that approved the study<br>• Include the approval number and/or a statement indicating approval of this research<br>• Indicate the form of consent obtained (written/oral) or the reason that consent was not obtained (e.g. the data were analyzed anonymously)<br><br>**Animal Research (involving vertebrate animals, embryos or tissues)**<br>• Provide the name of the Institutional Animal Care and Use Committee (IACUC) or other relevant ethics board that reviewed the study protocol, and indicate whether they approved this research or granted a formal waiver of ethical approval<br>• Include an approval number if one was obtained<br>• If the study involved *non-human primates*, add *additional details* about animal welfare and steps taken to ameliorate suffering<br>• If anesthesia, euthanasia, or any kind of animal sacrifice is part of the study, include briefly which substances and/or methods were applied<br><br>**Field Research**<br><br>Include the following details if this study involves the collection of plant, animal, or other materials from a natural setting:<br>• Field permit number<br>• Name of the institution or relevant body that granted permission	
**Data Availability**<br><br>Authors are required to make all data underlying the findings described fully available, without restriction, and from the time of publication. PLOS allows rare exceptions to address legal and ethical concerns. See the PLOS Data Policy and FAQ for detailed information.	Yes - all data are fully available without restriction

A Data Availability Statement describing where the data can be found is required at submission. Your answers to this question constitute the Data Availability Statement and **will be published in the article**, if accepted.<br><br>**Important:** Stating 'data available on request from the author' is not sufficient. If your data are only available upon request, select 'No' for the first question and explain your exceptional situation in the text box.<br><br>Do the authors confirm that all data underlying the findings described in their manuscript are fully available without restriction?	
**Describe where the data may be found in full sentences. If you are copying our sample text, replace any instances of XXX with the appropriate details.**<br><br>• If the data are **held or will be held in a public repository**, include URLs, accession numbers or DOIs. If this information will only be available after acceptance, indicate this by ticking the box below. For example: *All XXX files are available from the XXX database (accession number(s) XXX, XXX.).*<br>• If the data are all contained **within the manuscript and/or Supporting Information files**, enter the following: *All relevant data are within the manuscript and its Supporting Information files.*<br>• If neither of these applies but you are able to provide **details of access elsewhere**, with or without limitations, please do so. For example:<br><br>*Data cannot be shared publicly because of [XXX]. Data are available from the XXX Institutional Data Access / Ethics Committee (contact via XXX) for researchers who meet the criteria for access to confidential data.*<br><br>*The data underlying the results presented in the study are available from (include the name of the third party*	All relevant data are within the manuscript and its Supporting Information files.

and contact information or URL).<br>• This text is appropriate if the data are owned by a third party and authors do not have permission to share the data.<br><br>* typeset	
Additional data availability information:	Tick here if the URLs/accession numbers/DOIs will be available only after acceptance of the manuscript for publication so that we can ensure their inclusion before publication.

Powered by Editorial Manager® and ProduXion Manager® from Aries Systems Corporation

Manuscript

Click here to access/download;Manuscript;plos one Manuscript 109.docx

Title page

Molecular characterization and antibiotic resistance of *Acinetobacter baumannii* in cerebrospinal fluid and blood

Short title: **Epidemiology of *Acinetobacter baumannii* in Cerebrospinal Fluid and Blood**

Xiaohong Shi[1†], Hong Wang[1†], Xin Wang[2], Huaiqi Jing[2], Ran Duan[2], Shuai Qin[2], Dongyue Lv[2], Yufeng Fan[2], Zhenzhou Huang[2], B. Kyle Stirling[4, 5], Lei Zhang[3, 4], Jiazheng Wang[1*]

Author's affiliation

1 Department of Clinical Laboratory Shandong Provincial Qianfoshan Hospital, the First Affiliated Hospital of Shandong First Medical University, Jinan, Shandong, China.

2 State Key Laboratory for Infectious Disease Prevention and Control, National Institute for Communicable Disease Control and Prevention, Changping, Beijing, 102206, People's Republic of China.

3 Institute for Medical Dataology, Cheeloo College of Medicine, Shandong University, Jinan, China

4 Department of Biostatistics, School of Public Health, Cheeloo College of Medicine, Shandong University, Jinan, China

5 Luddy School of Informatics, Computing and Engineering, Indiana University, Bloomington, IN, USA

Corresponding author

* E-mail: wangjiazheng1210@163.com

† These authors contributed equally to this work

Abstract

To identify the antimicrobial resistance, carbapenemases and genetic relatedness of *A. baumannii* isolates from cerebrospinal fluid (CSF) and blood, a total of 50 nonrepetitive CSF *A. baumannii* isolates and 44 blood isolates were collected. The resistance phenotypes were determined according to the Clinical and Laboratory Standards Institute guidelines. We performed Polymerase Chain Reaction (PCR) experiments to detect the carbapenem resistance mechanism. Finally, we conducted Multilocus Sequence Typing (MLST) to depict the genetic relatedness of these isolates. We observed that eighty-eight of the 94 isolates collected were resistant to imipenem or meropenem. Among them, the *bla*$_{OXA-23}$ gene was the most prevalent carbapenemase gene with a 91.5% (86/94) detection rate, followed by the *bla*$_{OXA-24}$ gene that showed a 2.1% (2/94) detection rate isolates. Among all CRAB observations in this study, isolates with the *bla*$_{OXA-23}$ gene were resistant to both imipenem and meropenem. However, isolates positive for the *bla*$_{OXA-24}$ gene but negative for the *bla*$_{OXA-23}$ gene showed an imipenem-sensitive but meropenem-resistant phenotype. The outcome of multilocus sequence typing analysis showed 21 different STs were distinguished, of which ST195 (25.5%), ST540 (12.8%) and ST208 (11.7%) were most frequently observed. Eighty of the 94 isolates (85.1%) were clustered into CC92, and all CC92 isolates showed a carbapenem resistance phenotype (except AB13). Five novel STs were detected, and most of them were CRAB. In conclusion, a high level of carbapenem resistance was detected in this study. The CC92 and *bla*$_{OXA-23}$ gene were predominant. Five novel STs were detected, and these new STs require further investigation to understand the nature of and to prevent

outbreaks caused by *A. baumannii*. Our study provides additional observations and epidemiological data of CSF and blood *A. baumannii* strains, which may improve future infection control measures and aid in potential clinical treatment in hospitals and other clinical settings.

Introduction

Acinetobacter baumannii is a nonfermentative, Gram-negative opportunistic pathogen that often causes disease among immunocompromised patients [1]. *A. baumannii* is often found in hospitals causing a variety of nosocomial infectious diseases, including bloodstream infections, urinary tract infections, meningitis and wound infections [2]. In recent years, *A. baumannii* has become an important bacterium to identify when treating and controlling infectious diseases because of its remarkable ability to evolve extensive drug resistance to many antibiotics [3].

Some observations are referred to as Critical Values because these laboratory values may indicate an urgent and a life-threatening situation for patients where treatment protocols indicate immediate therapy must be initiated. This includes microbiology results identifying the bacteria in cerebrospinal fluid (CSF) and blood [4, 5]. Therefore, the presence of *A. baumannii* strains in CSF and blood is a Critical Value to identify for practitioners as it can poses great difficulties for clinical treatment options.

Carbapenems are considered to be the most effective antibiotics against many multidrug-resistant bacteria [6]. However, the increase in carbapenem-resistant *Acinetobacter baumannii* (CRAB) isolates has recently become a global concern. CHINET surveillance data showed that from 2005 to 2018, the resistance of *A. baumannii* to imipenem and meropenem increased approximately twofold [7, 8]. The production of carbapenemase is one of the most common and important mechanisms for *A. baumannii* resistance to carbapenems. OXA-type (mainly OXA-23, OXA-24, OXA-48, OXA-51 and OXA-58) was the most prevalent

carbapenemases. Among them, OXA-23, OXA-24, OXA-48 and OXA-58 are acquired carbapenemases, whereas OXA-51 is intrinsic to *A. baumannii* strains [9-11]. In addition, because the coding genes are located in transferable genetic elements and can spread among *A. baumannii* and even into other bacteria [12, 13], New Delhi metallo-β-lactamase (NDM) and *Klebsiella pneumoniae* carbapenemase (KPC) producers have also shown significant importance for worldwide prevalence [14, 15].

The multilocus sequence typing (MLST) method has been widely used to depict genetic relatedness and for molecular epidemiological studies of *A. baumannii* [16, 17]. Previous molecular epidemiological studies have shown that CC92 is highly prevalent throughout China, and it is the clonal complex (CC) with the widest global distribution [18, 19].

In this work, we observe, investigate and characterized 94 *A. baumannii* isolates obtained from CSF and blood we acquired from patients in university hospital in Shandong, China between 2014 and 2019.

Materials and Methods

Ethical statement

As this study just focus on bacteria alone and does not use any human material or patient information, the Ethics Committee of The First Affiliated Hospital of Shandong First Medical University exempted this study from review and the Review Board also waived the need for informed consent.

Bacterial isolates

A total of 94 nonrepetitive *A. baumannii* isolates were obtained from CSF or blood samples of patients from different departments (neurosurgery, ICU, hematopathology and other wards) at the First Affiliated Hospital of Shandong First Medical University (Shandong, China). These samples were obtained from 2014 to 2019. All isolates were identified using MALDI-TOF MS (Bruker) and further verified by PCR products of 16S rDNA sequencing [20]. PCR products were sequenced by Tsingke BioTech Co., Ltd., followed by sequence alignment on the NCBI database.

Antimicrobial susceptibility test

All *A. baumannii* strains were tested for susceptibility to 14 antibiotics, including ticarcillin/clavulanic acid, piperacillin/tazobactam, ceftazidime, cefoperazone/sulbactam, cefepime, imipenem, meropenem, tobramycin, ciprofloxacin, levofloxacin, minocycline, tigecycline, colistin and trimethoprim/sulfamethoxazole by using a Vitek 2 compact system (bioMérieux, Marcy, France) with AST-N-335 cards. The results were evaluated according to

the Clinical and Laboratory Standards Institute (CLSI) criteria except for tigecycline were adapted from the United States Food and Drug Administration breakpoints.

PCR Experiments

PCR assays were carried out using conventional PCR amplification. The target genes included the *bla*$_{OXA-51}$, *bla*$_{OXA-23}$, *bla*$_{OXA-24}$, *bla*$_{OXA-58}$, *bla*$_{OXA-48}$, *bla*$_{NDM-1}$, and *bla*$_{KPC}$ genes. Table 1 shows the sequences used for primer design and the annealing temperatures. The reaction conditions of the PCR programs consisted of an initial elongation at 94°C for 5 minutes; followed by 30 cycles of 94°C for 30 seconds, the respective annealing temperatures (Table 1) for 30 seconds, and 72°C for 1 minute; and a final extension step at 72°C for 10 minutes. Positive amplicons were randomly selected for sequencing to verify the amplicons sequences.

Table 1| Primers used with their respective annealing temperatures.

Primer	Sequence	Amplicon length	Annealing temp	Ref
bla_{OXA-51}-F	5'-ATGAACATTAAAGCACTC-3'	353bp	46°C	[21]
bla_{OXA-51}-R	5'-CTATAAAATACCTAATTGTTC-3'			
bla_{OXA-23}-F	5'-GATCGGATTGGAGAACCAGA-3'	501bp	53°C	[21]
bla_{OXA-23}-R	5'-ATTTCTGACCGCATTTCCAT-3'			
bla_{OXA-24}-F	5'-GGTTAGTTGGCCCCCTTAAA-3'	246bp	53°C	[21]
bla_{OXA-24}-R	5'-AGTTGAGCGAAAAGGGGATT-3'			
bla_{OXA-58}-F	5'-AAGTATTGGGGCTTGTGCTG-3'	599bp	53°C	[21]
bla_{OXA-58}-R	5'-CCCCTCTGCGCTCTACATAC-3'			
bla_{KPC}-F	5'-GCTCAGGCGCAACTGTAAGT-3'	823bp	55°C	[22]
bla_{KPC}-R	5'-GTCCAGACGGAACGTGGTAT-3'			
bla_{NDM-1}-F	5'-TCTCGACATGCCGGGTTTCGG-3'	475bp	55°C	[23]
bla_{NDM-1}-R	5'-ACCGAGATTGCCGAGCGACTT-3			
bla_{OXA-48}-F	5'-GCGTGGTTAAGGATGAACAC-3'	438bp	52°C	[24]
bla_{OXA-48}-R	5'-CATCAAGTTCAACCCAACCG-3'			

Multilocus Sequence Typing (MLST)

MLST analyses were performed using the Oxford scheme [16], publicly available from the https://pubmlst.org/abaumannii/info/primers_Oxford.shtml. This MLST scheme is based

on the sequencing of fragments of seven housekeeping genes: citrate synthase (*gltA*), DNA gyrase subunit B (*gyrB*), glucose dehydrogenase B (*gdhB*), homologous recombination factor (*recA*), 60-kDa chaperonin (*cpn60*), glucose-6-phosphate isomerase (*gpi*), and RNA polymerase sigma factor (*rpoD*). Amplification reactions were carried out as described previously [19, 25], and sequencing was performed in both directions.

The sequences were aligned with the reference sequence from the MLST database (https://pubmlst.org/abaumannii/) using MEGA (version 4.0). BioEdit (version 7.0.1) was used to determine the allele assignments of the housekeeping genes before composing a profile of each strain. The newly identified STs were submitted to the MLST database curator for approval, and a number was assigned. A minimum-spanning tree using the allelic difference between isolates of the seven housekeeping genes was constructed using BioNumerics (Applied Math).

Results

Distribution of bacterial isolates

A. baumannii isolates were collected from two different locations, CSF (n=50) and blood (n=44), from the First Affiliated Hospital of Shandong First Medical University. For the CSF isolates, one was collected in 2014, four in 2015, seventeen in 2016, fifteen in 2017, five in 2018 and eight in 2019. Forty-one of these isolates were obtained from neurosurgery, six from the ICU and three from other wards. Regarding the *A. baumannii* isolates samples obtained from blood; twelve were collected in 2016, thirteen in 2017, eleven in 2018 and eight in 2019. Twenty-eight blood isolates were obtained from the ICU, six from neurosurgery, four from hematopathology and six from other wards.

Antibiotic susceptibility

The antimicrobial susceptibility profiles of 94 *A. baumannii* strains are shown in Table 2, Fig.1 and S1 Table in Supplemental Material. In general, more than 90% of the *A. baumannii* isolates were resistant to ticarcillin/clavulanic acid, piperacillin/tazobactam, ceftazidime and ciprofloxacin. 88 of 94 *A. baumannii* isolates were carbapenem resistance *Acinetobacter baumannii* (CRAB) isolates. Among them, 86 isolates were resistant to both imipenem and meropenem. Two isolates showed a meropenem-resistant but imipenem-sensitive phenotype. Most isolates (over 89%) were also resistant to these six antibiotics every year from 2016 to 2019 (Fig.1) (As only one strain was isolated in 2014 and only four strains were isolated in 2015, the analysis results were not included these two years). A total of 77.7% of the isolates

were resistant to tobramycin, 77.7% were resistant to levofloxacin, 59.6% to trimethoprim/sulfamethoxazole, 52.1% to cefepime and 45.7% to cefoperazone/sulbactam. However, the resistance rates for levofloxacin, trimethoprim/sulfamethoxazole and cefoperazone/sulbactam were increased year by year from 2017 to 2019. In contrast, the resistance rates for minocycline and tigecycline were only 19.1% and 7.4%, and were decreased year by year from 2017 to 2019. All isolates were sensitive to colistin. No statistical significances were observed between CSF and blood isolates in all antibiotics tested in this study.

Fig.1 Antibiotic resistance rates from 2016 to 2019

T/C: ticarcillin/clavulanic acid; P/T: piperacillin/tazobactam; CAZ: ceftazidime; CSL: cefoperazone/sulbactam; FEP: cefepime; IPM: imipenem; MEM: meropenem; TOB: tobramycin; CIP: ciprofloxacin; LEV: levofloxacin; MNO: minocycline; TGC: tigecycline; COL: colistin; SXT: sulfamethoxazole/trimethoprim.

Table 2| Resistance rates of *A. baumannii* isolates obtained from CSF and blood

Antimicrobial	Resistance rate (%)			P-value
	Overall (n = 94)	CSF (n = 50)	Blood (n = 44)	
ticarcillin/clavulanic acid	87（92.6）	47（94.0）	40（90.9）	0.569
piperacillin/tazobactam	87（92.6）	47（94.0）	40（90.9）	0.569
ceftazidime	87（92.6）	47（94.0）	40（90.9）	0.569
cefoperazone/sulbactam	43（45.7）	19（38.0）	24（54.5）	0.108
cefepime	49（52.1）	28（56.0）	21（47.7）	1.000
imipenem	86（91.5）	46（92.0）	40（90.9）	0.850
meropenem	88（93.6）	47（94.0）	41（93.2）	0.871
tobramycin	73（77.7）	41（82.0）	32（72.7）	0.281
ciprofloxacin	87（92.6）	47（94.0）	40（90.9）	0.569
levofloxacin	71（75.5）	38（76.0）	33（75.0）	0.910
minocycline	18（19.1）	6（12.0）	12（27.3）	0.060
tigecycline	7（7.4）	3（6.0）	4（9.1）	0.569

colistin	0（0）	0（0）	0（0）	-
trimethoprim/sulfamethoxazole	56（59.6）	34（68.0）	22（50.0）	0.076

Distribution of carbapenem resistance genes

All isolates were screened for the presence of carbapenem resistance genes (Table 3). In the 94 *A. baumannii* isolates, all of them harbored the intrinsic $bla_{\text{OXA-51}}$ gene. In contrast, none of them had $bla_{\text{OXA-48}}$, $bla_{\text{OXA-58}}$, $bla_{\text{NDM-1}}$ or bla_{KPC} genes. The $bla_{\text{OXA-23}}$ gene was the most prevalent carbapenemase gene with a 91.5% (86/94) detection rate, followed by the $bla_{\text{OXA-24}}$ gene in only 2 (2.1%) isolates. No statistical significances were observed between CSF and blood isolates for all carbapenem resistance genes detected.

In this study, all of the isolates with $bla_{\text{OXA-23}}$ or $bla_{\text{OXA-24}}$ genes were carbapenem resistant. Among them, isolates with the $bla_{\text{OXA-23}}$ gene were resistant to both imipenem and meropenem. However, isolates positive for the $bla_{\text{OXA-24}}$ gene but negative for the $bla_{\text{OXA-23}}$ gene showed an imipenem-sensitive but meropenem-resistant phenotype.

Table 3 | Prevalence of carbapenem resistance genes

Gene	No. of positive isolates (% of 94)	No. distributing in CSF (n = 50) n (%)	No. distributing in Blood (n = 44) n (%)	P-value
$\mathit{bla}_{\text{OXA-23}}$	86(91.5)	46 (92.0)	40 (90.9)	0.850
$\mathit{bla}_{\text{OXA-24}}$	2(2.1)	1 (2.0)	1 (2.3)	0.927
$\mathit{bla}_{\text{OXA-48}}$	0	0 (0)	0 (0)	-
$\mathit{bla}_{\text{OXA-51}}$	94(100)	50 (100)	44 (100)	-
$\mathit{bla}_{\text{OXA-58}}$	0	0 (0)	0 (0)	-
$\mathit{bla}_{\text{NDM-1}}$	0	0 (0)	0 (0)	-
$\mathit{bla}_{\text{KPC}}$	0	0 (0)	0 (0)	-

MLST profile

The MLST analysis revealed a total of 21 different STs, including 16 existing STs and 5 novel STs we identified in this study (the new STs were submitted for ST assignment which were ST1967, ST1968, ST1969, ST1970 and ST1971). The profiles of the newly identified ST types are listed in Table 4. Among them, 89.4% (84/94) were represented by 11 main STs (having ≥2 isolates), and the prevalent STs were ST195, ST540 and ST208, accounting for 25.5% (24/94), 12.8% (12/94), and 11.7% (11/94), respectively (Fig.2 and S2 Table in the

supplemental material).

Fig.2 Minimum spanning tree of 94 *Acinetobacter baumannii* isolates from cerebrospinal fluid and blood based on MLST. Each ST is represented by a circle sized in proportion to the number of isolates represented by that ST, isolates within the pink shaded area are the CC92 isolates, and the number of allelic difference between STs is indicated on the branches. The detailed MLST profiles can be seen in S2 Table in Supplementary Material.

Table 4 | Allelic profiles of the new STs found in this study

STs	*gltA*	*gyrB*	*gdhB*	*recA*	*cpn60*	*gpi*	*rpoD*
1967	1	34	3	2	2	178	3
1968	1	3	3	2	2	113	3
1969	1	17	135	12	23	98	6
1970	1	3	3	2	2	160	4
1971	36	34	59	28	4	279	3

ST 195 was also the dominant ST in 2016, 2017 and 2019. However, in 2018, this ST didn't appear and the dominant ST was changed to ST540 and ST191. ST540 and ST208 isolates could be found in every year from 2016 to 2019 but the ratio changed year by year (Fig.3). Although none of the ST191 strain was found in CSF and blood from 2014 to 2016, it was emerged in 2017 and even rose to 25% in 2018. For other minor ST types, the distributions were different in different years. For instance, the ratio of ST369 isolates increased from 3.4% in 2016 to 10.7% in 2017. In 2018, this type of strain didn’t appear but the ratio rose to 12.5% in 2019 (As only one strain was isolated in 2014 and only four strains

were isolated in 2015, the analysis results were not included these two years).

Fig.3 MLST population analysis over the different years. (A) STs in all 94 isolates. (B) STs distribution in 2016. (C) STs distribution in 2017. (D) STs distribution in 2018. (E) STs distribution in 2019.

Twelve STs representing 85.1% (80/94) of the isolates were clustered into CC92, with up to 18 different allelic profiles and 56 different isolates being represented. In addition, 9 individual STs accounted for 14 isolates (The detailed MLST profiles can be seen in S2 Table in Supplemental Material).

MLST, antibiotic susceptibility and carbapenem resistance genes

All of the isolates grouped into CC92 were carbapenem-resistant *A. baumannii* except for one isolate (AB13). These isolates were also not sensitive to ticarcillin/clavulanic acid, piperacillin/tazobactam, ceftazidime, cefepime, ciprofloxacin and levofloxacin but had variable susceptibilities to cefoperazone/sulbactam, tobramycin, minocycline, tigecycline and trimethoprim/sulfamethoxazole. In contrast, all of the CSAB isolates belonged to individual STs except for one isolate (AB13). These isolates were also sensitive to the other 12 antibiotics tested in this study.

Five novel STs were identified in this study. All 4 ST1967 were isolated from CSF and were non-sensitive to all β-lactam antibiotics and quinolones. For ST 1968 isolates, 5 were non-sensitive to 13 antibiotics (except colistin) tested in this study, but the other one isolate

(AB73) was sensitive to tobramycin, trimethoprim/sulfamethoxazole and colistin. ST1970 isolate was non-sensitive to 12 antibiotics (except tigecycline and colistin) tested in this study, whereas the ST1971 isolate was sensitive to all antibiotics. ST1969 contained two isolates (AB29 and AB70). Both of these isolates possessed the *bla*$_{OXA-24}$ gene and were resistant to meropenem but sensitive to imipenem.

Bla$_{OXA-23}$ gene was more prevalent than *bla*$_{OXA-24}$ gene but the detection rates altered among different STs. All isolates in CC92 (contain 12 STs) carried *bla*$_{OXA-23}$ gene (except only one isolate in ST208). In contrast, *bla*$_{OXA-23}$ gene could be detected in only 50% (7/14) isolates of individual STs. None of the isolates in ST373, ST377, ST712, ST1969 and ST1971 possessed this gene.

Discussion

CSF and blood infection of *A. baumannii* (especially for CRAB) may be life-threatening and bring great obstacles for clinical treatment [4, 5]. This study offers insight into the molecular characterization and antibiotic resistance of *A. baumannii* from CSF and blood.

CHINET surveillance data showed that, in China, from 2005 to 2018, the resistance rate of *A. baumannii* for imipenem and meropenem increased from 32.9% to 71.7% and from 41.3% to 78.1%, respectively [7, 8]. Compared to the surveillance data, the resistance rates of *A. baumannii* for imipenem and meropenem in our experiment were 91.5% and 93.6%, respectively, more than twofold higher than the surveillance data in 2005 and more than 10% higher than 2018. For CSF and blood infection, the recommended dose of antibiotics was usually higher and needed a longer course of treatment than the case of superficial infection. However the CHINET surveillance data contained antibiotic resistance data for bacteria isolated from various sites, including some superficial infections. This might be the cause of the higher drug resistance rate in our study. In addition, the CHINET surveillance data [7, 8, 26, 27] demonstrated that the resistance rate of *A. baumannii* to meropenem is slightly higher than that to imipenem. This is similar to our observations and results. In fact, our results also support the view that imipenem is more bactericidal [28] and has a higher T>MIC value [29] than meropenem against *A. baumannii*. In contrast, the resistance rate to tigecycline was only 7.4%, and all isolates showed colistin sensitive phenotype. The resistance rates of these two drugs were far below carbapenem and the other antibiotics tested in this study, suggesting that these two drugs might serve as therapeutic agents to control infections.

To investigate the mechanism of carbapenem resistance, carbapenemase-encoding genes were tested in this study. Our results showed that the *bla*$_{OXA-23}$ gene existed in most CRAB isolates but was absent for all CSAB isolates, which indicated that *bla*$_{OXA-23}$ was the major mechanism for carbapenem resistance of CRAB isolated from CSF and blood. The *bla*$_{OXA-23}$ gene was also the most important mechanism for carbapenem resistance in CRAB in China [19, 25] and some other countries [30-32]. On the other hand, the *bla*$_{OXA-24}$ gene was another mechanism for carbapenem resistance, as *bla*$_{OXA-24}$-positive but *bla*$_{OXA-23}$-negative isolates in this study showed meropenem-resistance and imipenem-sensitive characteristics. *Bla*$_{OXA-24}$-positive *A. baumannii* strains have been reported in many countries [11, 33, 34], especially in Spain, where *bla*$_{OXA-24}$ was the most prevalent gene [11]. Interestingly, even though most *bla*$_{OXA-24}$-positive isolates have been reported to be resistant to both imipenem and meropenem, in our experiment, *bla*$_{OXA-24}$-positive strains showed an imipenem-sensitive but meropenem-resistant phenotype. Some molecular biological mechanisms have been reported in many Gram-negative bacteria to explain the imipenem-sensitive but meropenem-resistant phenomenon. For example, the transmission of the *bla*$_{IMP-6}$ and *bla*$_{CTX-M-2}$ plasmids [35] as well as the absence of OmpK35 and the frame shift mutation in OmpK36 [36] have been shown to be important mechanisms for imipenem-sensitive but meropenem-resistant *Klebsiella pneumoniae* (ISMRKP) strains. Substrate specificities of efflux pumps lead to different drug resistance characteristics for *Pseudomonas aeruginosa*. As a specific substrate, meropenem could be extruded by many efflux pumps, but imipenem was not affected by these efflux systems [37]. As a result, some imipenem-sensitive but

meropenem-resistant *Pseudomonas aeruginosa* strains were detected. However, studies examining this mechanism against *A. baumannii* are scarce. Both of these strains belonged to a novel ST (ST1969). This molecular mechanism for *A. baumannii* strains will require further investigation.

ST540, ST195 and ST208 were three major STs for *A. baumannii* isolated from CSF and blood. Among them, ST195 and ST208 are two dominant STs currently found in China [38-40]. In this study, ST 195 was also the dominant ST in 2016, 2017 and 2019. However, no ST195 isolates could be found in 2018. Although ST540 was not the main ST observed in China, it was shown that ST540 was not only one of the three common STs but also the predicted founder of the CC for *A. baumannii* isolated from blood and CSF. ST208 isolates were detected every year from 2016 to 2019, but the ratio changed year by year. On a global scale, CC92 was the largest and most geographically diverse CC, which was widespread in many countries [38], including China [25]. Although we did not find ST92 isolates (the predicted founder of CC92) in this study, it was shown that most *A. baumannii* isolated from CSF and blood belonged to CC92 (CC92 was the unique CC clonal group tested). In addition, 79 of 80 CC92 isolates were CRAB (except for one isolate, AB13), whereas only approximately 50% of the individual STs isolates were resistant to imipenem or meropenem, which suggested that CC92 isolates tend to acquire carbapenem resistance determinants. A previous study reported that CC92 is a widespread variant that has advantages in acquiring resistance determinants and surviving in the nosocomial environment, which renders it preferentially selected under antibiotic pressure [19]. This is a possible reason for the high

detection rate of CC92 *A. baumannii* isolates in CSF and blood and the high correlation between CC92 and carbapenem resistance characteristics.

We also observed a total of five new STs. Among them, two novel STs were classified into CC92 and others were individual STs, which suggested that *A. baumannii* isolates were diverse and still in clonal expansion. As 13 of the 14 isolates in the five new STs were identified as CRAB, close attention should be paid toward these new STs to identify and further limit both transmission and outbreaks.

Conclusions

In summary, with our study, we described the molecular characterization and antibiotic resistance of *A. baumannii* from CSF and blood in a hospital in Shandong, China. A high level of carbapenem resistance was detected. The majority of the isolates was CRAB, carried the bla_{OXA-23} carbapenemase gene and belonged to MLST CC92. Five novel STs were detected, and most of them were CRAB, some of which belonged to CC92. This study offers new epidemiological data of CSF and blood *A. baumannii* strains, which may help to improve infection control measures and clinical treatment in hospitals.

Acknowledgements

We thank Duochun Wang and Tao Xiao for some helpful comments on our manuscript.

References

1. Hu Y, He L, Tao X, Meng F, Zhang J. Biofilm may not be Necessary for the Epidemic Spread of Acinetobacter baumannii. Scientific reports. 2016;6:32066. Epub 2016/08/26. doi: 10.1038/srep32066. PubMed PMID: 27558010; PubMed Central PMCID: PMCPMC4997352.
2. Peleg AY, Seifert H, Paterson DL. Acinetobacter baumannii: emergence of a successful pathogen. Clinical microbiology reviews. 2008;21(3):538-82. Epub 2008/07/16. doi: 10.1128/cmr.00058-07. PubMed PMID: 18625687; PubMed Central PMCID: PMCPMC2493088.
3. Fournier PE, Vallenet D, Barbe V, Audic S, Ogata H, Poirel L, et al. Comparative genomics of multidrug resistance in Acinetobacter baumannii. PLoS genetics. 2006;2(1):e7. Epub 2006/01/18. doi: 10.1371/journal.pgen.0020007. PubMed PMID: 16415984; PubMed Central PMCID: PMCPMC1326220.
4. Chapman CN, Otis CN. From critical values to critical diagnoses: a review with an emphasis on cytopathology. Cancer cytopathology. 2011;119(3):148-57. Epub 2011/04/22. doi: 10.1002/cncy.20158. PubMed PMID: 21509947.
5. Howanitz PJ, Steindel SJ, Heard NV. Laboratory critical values policies and procedures: a college of American Pathologists Q-Probes Study in 623 institutions. Archives of pathology & laboratory medicine. 2002;126(6):663-9. Epub 2002/05/30. doi: 10.1043/0003-9985(2002)126<0663:lcvpap>2.0.co;2. PubMed PMID: 12033953.
6. Biglari S, Alfizah H, Ramliza R, Rahman MM. Molecular characterization of

carbapenemase and cephalosporinase genes among clinical isolates of Acinetobacter baumannii in a tertiary medical centre in Malaysia. Journal of medical microbiology. 2015;64(Pt 1):53-8. Epub 2014/11/09. doi: 10.1099/jmm.0.082263-0. PubMed PMID: 25381148.

7. Hu FP, Guo Y, Zhu DM, Wang F, Jiang XF, Xu YC, et al. Resistance trends among clinical isolates in China reported from CHINET surveillance of bacterial resistance, 2005-2014. Clinical microbiology and infection : the official publication of the European Society of Clinical Microbiology and Infectious Diseases. 2016;22 Suppl 1:S9-14. Epub 2016/03/24. doi: 10.1016/j.cmi.2016.01.001. PubMed PMID: 27000156.

8. Hu F, Guo Y, Yang Y, Zheng Y, Wu S, Jiang X, et al. Resistance reported from China antimicrobial surveillance network (CHINET) in 2018. European journal of clinical microbiology & infectious diseases : official publication of the European Society of Clinical Microbiology. 2019;38(12):2275-81. Epub 2019/09/04. doi: 10.1007/s10096-019-03673-1. PubMed PMID: 31478103.

9. Evans BA, Amyes SG. OXA β-lactamases. Clinical microbiology reviews. 2014;27(2):241-63. Epub 2014/04/04. doi: 10.1128/cmr.00117-13. PubMed PMID: 24696435; PubMed Central PMCID: PMCPMC3993105.

10. Nowak P, Paluchowska P. Acinetobacter baumannii: biology and drug resistance - role of carbapenemases. Folia histochemica et cytobiologica. 2016;54(2):61-74. Epub 2016/06/09. doi: 10.5603/FHC.a2016.0009. PubMed PMID: 27270503.

11. Dahdouh E, Gómez-Gil R, Pacho S, Mingorance J, Daoud Z, Suárez M. Clonality,

virulence determinants, and profiles of resistance of clinical Acinetobacter baumannii isolates obtained from a Spanish hospital. PloS one. 2017;12(4):e0176824. Epub 2017/04/28. doi: 10.1371/journal.pone.0176824. PubMed PMID: 28448572; PubMed Central PMCID: PMCPMC5407824.

12. Pagano M, Martins AF, Barth AL. Mobile genetic elements related to carbapenem resistance in Acinetobacter baumannii. Brazilian journal of microbiology : [publication of the Brazilian Society for Microbiology]. 2016;47(4):785-92. Epub 2016/08/16. doi: 10.1016/j.bjm.2016.06.005. PubMed PMID: 27522927; PubMed Central PMCID: PMCPMC5052331.

13. Partridge SR, Kwong SM, Firth N, Jensen SO. Mobile Genetic Elements Associated with Antimicrobial Resistance. Clinical microbiology reviews. 2018;31(4). Epub 2018/08/03. doi: 10.1128/cmr.00088-17. PubMed PMID: 30068738; PubMed Central PMCID: PMCPMC6148190.

14. Chen Y, Zhou Z, Jiang Y, Yu Y. Emergence of NDM-1-producing Acinetobacter baumannii in China. The Journal of antimicrobial chemotherapy. 2011;66(6):1255-9. Epub 2011/03/15. doi: 10.1093/jac/dkr082. PubMed PMID: 21398294.

15. Azimi L, Talebi M, Pourshafie MR, Owlia P, Rastegar Lari A. Characterization of Carbapenemases in Extensively Drug Resistance Acinetobacter baumannii in a Burn Care Center in Iran. International journal of molecular and cellular medicine. 2015;4(1):46-53. Epub 2015/03/31. PubMed PMID: 25815282; PubMed Central PMCID: PMCPMC4359705.

16. Bartual SG, Seifert H, Hippler C, Luzon MA, Wisplinghoff H, Rodríguez-Valera F. Development of a multilocus sequence typing scheme for characterization of clinical isolates of Acinetobacter baumannii. Journal of clinical microbiology. 2005;43(9):4382-90. Epub 2005/09/08. doi: 10.1128/jcm.43.9.4382-4390.2005. PubMed PMID: 16145081; PubMed Central PMCID: PMCPMC1234098.

17. Diancourt L, Passet V, Nemec A, Dijkshoorn L, Brisse S. The population structure of Acinetobacter baumannii: expanding multiresistant clones from an ancestral susceptible genetic pool. PloS one. 2010;5(4):e10034. Epub 2010/04/13. doi: 10.1371/journal.pone.0010034. PubMed PMID: 20383326; PubMed Central PMCID: PMCPMC2850921.

18. Runnegar N, Sidjabat H, Goh HM, Nimmo GR, Schembri MA, Paterson DL. Molecular epidemiology of multidrug-resistant Acinetobacter baumannii in a single institution over a 10-year period. Journal of clinical microbiology. 2010;48(11):4051-6. Epub 2010/08/27. doi: 10.1128/jcm.01208-10. PubMed PMID: 20739495; PubMed Central PMCID: PMCPMC3020849.

19. Ruan Z, Chen Y, Jiang Y, Zhou H, Zhou Z, Fu Y, et al. Wide distribution of CC92 carbapenem-resistant and OXA-23-producing Acinetobacter baumannii in multiple provinces of China. International journal of antimicrobial agents. 2013;42(4):322-8. Epub 2013/08/31. doi: 10.1016/j.ijantimicag.2013.06.019. PubMed PMID: 23988720.

20. Hao H, Liang J, Duan R, Chen Y, Liu C, Xiao Y, et al. Yersinia spp. Identification Using Copy Diversity in the Chromosomal 16S rRNA Gene Sequence. PloS one.

2016;11(1):e0147639. Epub 2016/01/26. doi: 10.1371/journal.pone.0147639. PubMed PMID: 26808495; PubMed Central PMCID: PMCPMC4726496.

21. Woodford N, Ellington MJ, Coelho JM, Turton JF, Ward ME, Brown S, et al. Multiplex PCR for genes encoding prevalent OXA carbapenemases in Acinetobacter spp. International journal of antimicrobial agents. 2006;27(4):351-3. Epub 2006/03/28. doi: 10.1016/j.ijantimicag.2006.01.004. PubMed PMID: 16564159.

22. Li Y, Guo Q, Wang P, Zhu D, Ye X, Wu S, et al. Clonal dissemination of extensively drug-resistant Acinetobacter baumannii producing an OXA-23 β-lactamase at a teaching hospital in Shanghai, China. Journal of microbiology, immunology, and infection = Wei mian yu gan ran za zhi. 2015;48(1):101-8. Epub 2014/05/28. doi: 10.1016/j.jmii.2014.04.005. PubMed PMID: 24863499.

23. Chang Y, Luan G, Xu Y, Wang Y, Shen M, Zhang C, et al. Characterization of carbapenem-resistant Acinetobacter baumannii isolates in a Chinese teaching hospital. Frontiers in microbiology. 2015;6:910. Epub 2015/09/22. doi: 10.3389/fmicb.2015.00910. PubMed PMID: 26388854; PubMed Central PMCID: PMCPMC4555021.

24. Poirel L, Walsh TR, Cuvillier V, Nordmann P. Multiplex PCR for detection of acquired carbapenemase genes. Diagnostic microbiology and infectious disease. 2011;70(1):119-23. Epub 2011/03/15. doi: 10.1016/j.diagmicrobio.2010.12.002. PubMed PMID: 21398074.

25. Fu Y, Zhou J, Zhou H, Yang Q, Wei Z, Yu Y, et al. Wide dissemination of

OXA-23-producing carbapenem-resistant Acinetobacter baumannii clonal complex 22 in multiple cities of China. The Journal of antimicrobial chemotherapy. 2010;65(4):644-50. Epub 2010/02/16. doi: 10.1093/jac/dkq027. PubMed PMID: 20154023.

26. Xiao YH, Giske CG, Wei ZQ, Shen P, Heddini A, Li LJ. Epidemiology and characteristics of antimicrobial resistance in China. Drug resistance updates : reviews and commentaries in antimicrobial and anticancer chemotherapy. 2011;14(4-5):236-50. Epub 2011/08/03. doi: 10.1016/j.drup.2011.07.001. PubMed PMID: 21807550.

27. Hu F, Zhu D, Wang F, Wang M. Current Status and Trends of Antibacterial Resistance in China. Clinical infectious diseases : an official publication of the Infectious Diseases Society of America. 2018;67(suppl_2):S128-S34. Epub 2018/11/14. doi: 10.1093/cid/ciy657. PubMed PMID: 30423045.

28. Novelli A, Adembri C, Livi P, Fallani S, Mazzei T, De Gaudio AR. Pharmacokinetic evaluation of meropenem and imipenem in critically ill patients with sepsis. Clinical pharmacokinetics. 2005;44(5):539-49. Epub 2005/05/06. doi: 10.2165/00003088-200544050-00007. PubMed PMID: 15871639.

29. Kuti JL, Florea NR, Nightingale CH, Nicolau DP. Pharmacodynamics of meropenem and imipenem against Enterobacteriaceae, Acinetobacter baumannii, and Pseudomonas aeruginosa. Pharmacotherapy. 2004;24(1):8-15. Epub 2004/01/27. doi: 10.1592/phco.24.1.8.34804. PubMed PMID: 14740783.

30. Merino M, Poza M, Roca I, Barba MJ, Sousa MD, Vila J, et al. Nosocomial outbreak of a multiresistant Acinetobacter baumannii expressing OXA-23 carbapenemase in Spain.

Microbial drug resistance (Larchmont, NY). 2014;20(4):259-63. Epub 2013/12/18. doi: 10.1089/mdr.2013.0127. PubMed PMID: 24328852.

31. Zowawi HM, Sartor AL, Sidjabat HE, Balkhy HH, Walsh TR, Al Johani SM, et al. Molecular epidemiology of carbapenem-resistant Acinetobacter baumannii isolates in the Gulf Cooperation Council States: dominance of OXA-23-type producers. Journal of clinical microbiology. 2015;53(3):896-903. Epub 2015/01/09. doi: 10.1128/jcm.02784-14. PubMed PMID: 25568439; PubMed Central PMCID: PMCPMC4390656.

32. El Bannah AMS, Nawar NN, Hassan RMM, Salem STB. Molecular Epidemiology of Carbapenem-Resistant Acinetobacter baumannii in a Tertiary Care Hospital in Egypt: Clonal Spread of blaOXA-23. Microbial drug resistance (Larchmont, NY). 2018;24(3):269-77. Epub 2017/08/08. doi: 10.1089/mdr.2017.0057. PubMed PMID: 28783427.

33. Castanheira M, Wanger A, Kruzel M, Deshpande LM, Jones RN. Emergence and clonal dissemination of OXA-24- and OXA-58-producing Acinetobacter baumannii strains in Houston, Texas: report from the SENTRY Antimicrobial Surveillance Program. Journal of clinical microbiology. 2008;46(9):3179-80. Epub 2008/09/05. doi: 10.1128/jcm.00988-08. PubMed PMID: 18768660; PubMed Central PMCID: PMCPMC2546771.

34. Todorova B, Velinov T, Ivanov I, Dobreva E, Kantardjiev T. First detection of OXA-24 carbapenemase-producing Acinetobacter baumannii isolates in Bulgaria. World journal

of microbiology & biotechnology. 2014;30(4):1427-30. Epub 2013/11/30. doi: 10.1007/s11274-013-1562-3. PubMed PMID: 24287943.

35. Kayama S, Shigemoto N, Kuwahara R, Ishino T, Imon K, Onodera M, et al. The first case of septicemia caused by imipenem-susceptible, meropenem-resistant Klebsiella pneumoniae. Annals of laboratory medicine. 2013;33(5):383-5. Epub 2013/09/05. doi: 10.3343/alm.2013.33.5.383. PubMed PMID: 24003434; PubMed Central PMCID: PMCPMC3756248.

36. Kayama S, Koba Y, Shigemoto N, Kuwahara R, Kakuhama T, Kimura K, et al. Imipenem-susceptible, meropenem-resistant Klebsiella pneumoniae producing OXA-181 in Japan. Antimicrobial agents and chemotherapy. 2015;59(2):1379-80. Epub 2014/12/17. doi: 10.1128/aac.04330-14. PubMed PMID: 25512424; PubMed Central PMCID: PMCPMC4335852.

37. Masuda N, Sakagawa E, Ohya S, Gotoh N, Tsujimoto H, Nishino T. Substrate specificities of MexAB-OprM, MexCD-OprJ, and MexXY-oprM efflux pumps in Pseudomonas aeruginosa. Antimicrobial agents and chemotherapy. 2000;44(12):3322-7. Epub 2000/11/18. doi: 10.1128/aac.44.12.3322-3327.2000. PubMed PMID: 11083635; PubMed Central PMCID: PMCPMC90200.

38. Karah N, Sundsfjord A, Towner K, Samuelsen Ø. Insights into the global molecular epidemiology of carbapenem non-susceptible clones of Acinetobacter baumannii. Drug resistance updates : reviews and commentaries in antimicrobial and anticancer chemotherapy. 2012;15(4):237-47. Epub 2012/07/31. doi: 10.1016/j.drup.2012.06.001.

PubMed PMID: 22841809.

39. Huang G, Yin S, Gong Y, Zhao X, Zou L, Jiang B, et al. Multilocus Sequence Typing Analysis of Carbapenem-Resistant Acinetobacter baumannii in a Chinese Burns Institute. Frontiers in microbiology. 2016;7:1717. Epub 2016/11/25. doi: 10.3389/fmicb.2016.01717. PubMed PMID: 27881972; PubMed Central PMCID: PMCPMC5101237.

40. Qu J, Du Y, Yu R, Lü X. The First Outbreak Caused by Acinetobacter baumannii ST208 and ST195 in China. BioMed research international. 2016;2016:9254907. Epub 2016/05/05. doi: 10.1155/2016/9254907. PubMed PMID: 27144176; PubMed Central PMCID: PMCPMC4842041.

Supporting information

S1 Table Minimum Inhibitory Concentrations (MICs) of the tested antimicrobial agents

T/C: ticarcillin/clavulanic acid; P/T: piperacillin/tazobactam; CAZ: ceftazidime; CSL: cefoperazone/sulbactam; FEP: cefepime; IPM: imipenem; MEM: meropenem; TOB: tobramycin; CIP: ciprofloxacin; LEV: levofloxacin; MNO: minocycline; TGC: tigecycline; COL: colistin; SXT: sulfamethoxazole/trimethoprim.

S2 Table Allelic profiles of the *A. baumannii* strains used in this study

Click here to access/download;Figure;Fig.1.jpg

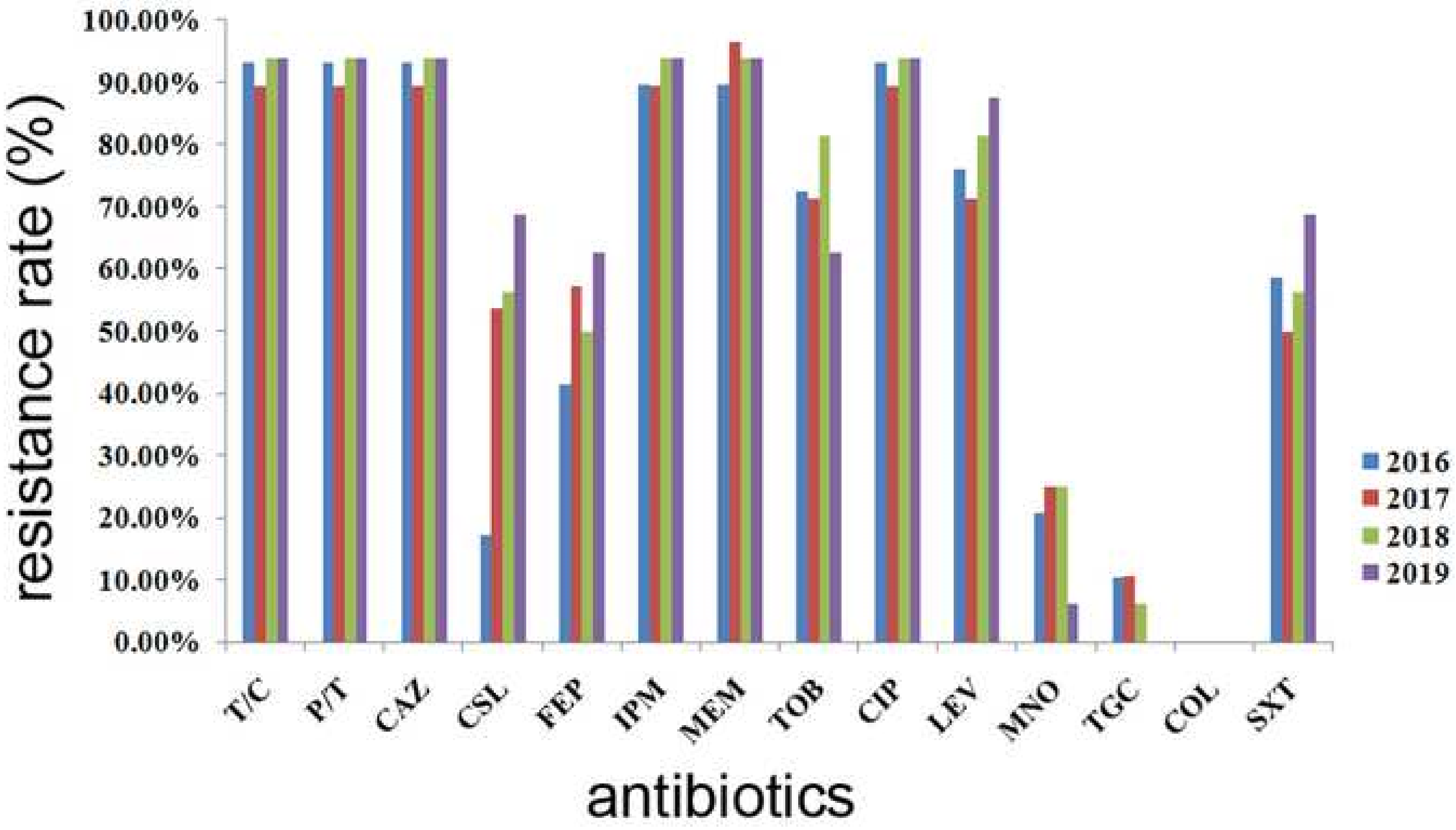

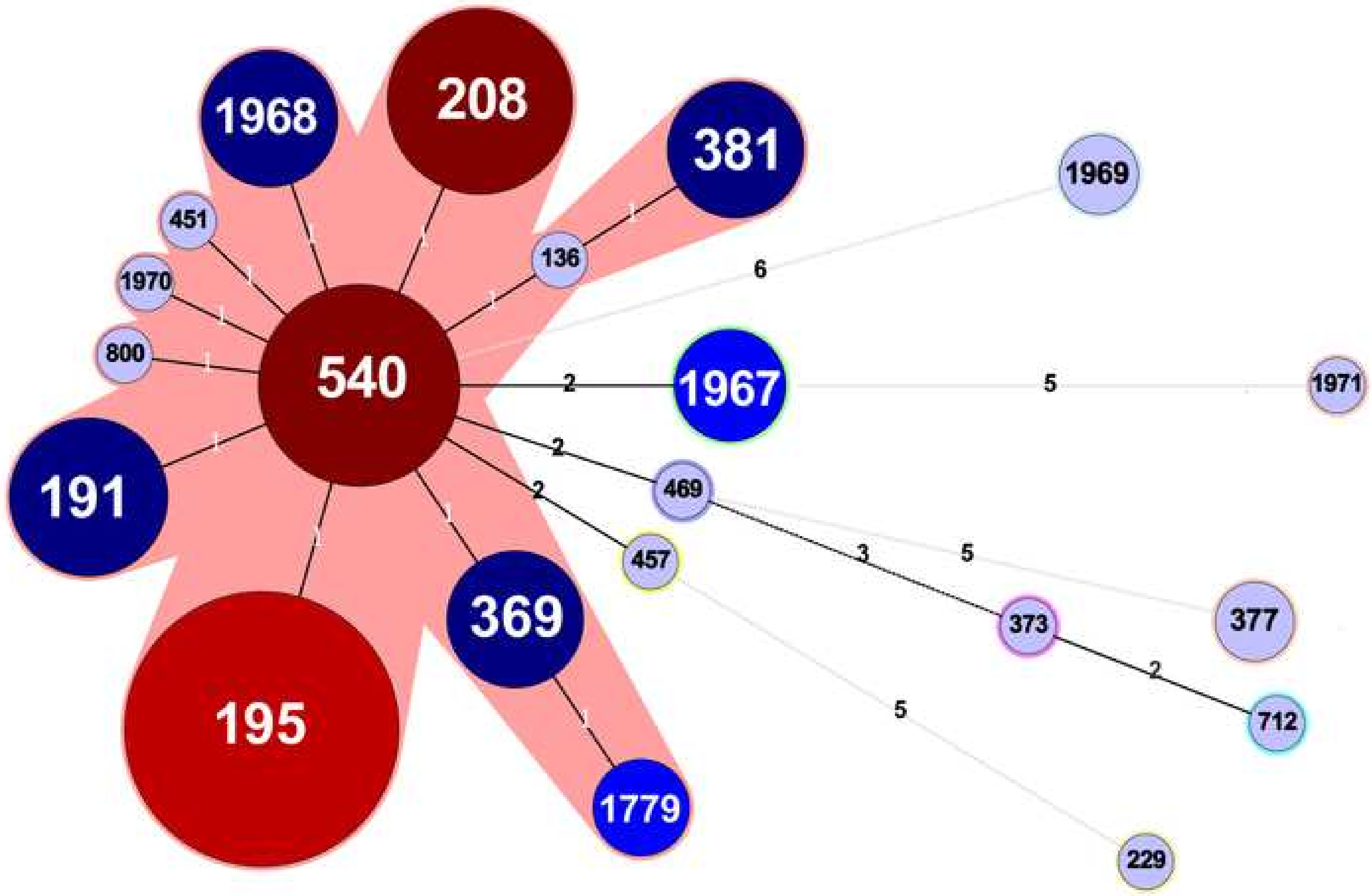
1968
208
381
1969
451
136
6
1970
800
540
2
1967
5
1971
191
2
469
2
457
3
5
377
373
369
2
712
195
5
1779
229

Figure

Click here to access/download;Figure;Fig.3.jpg

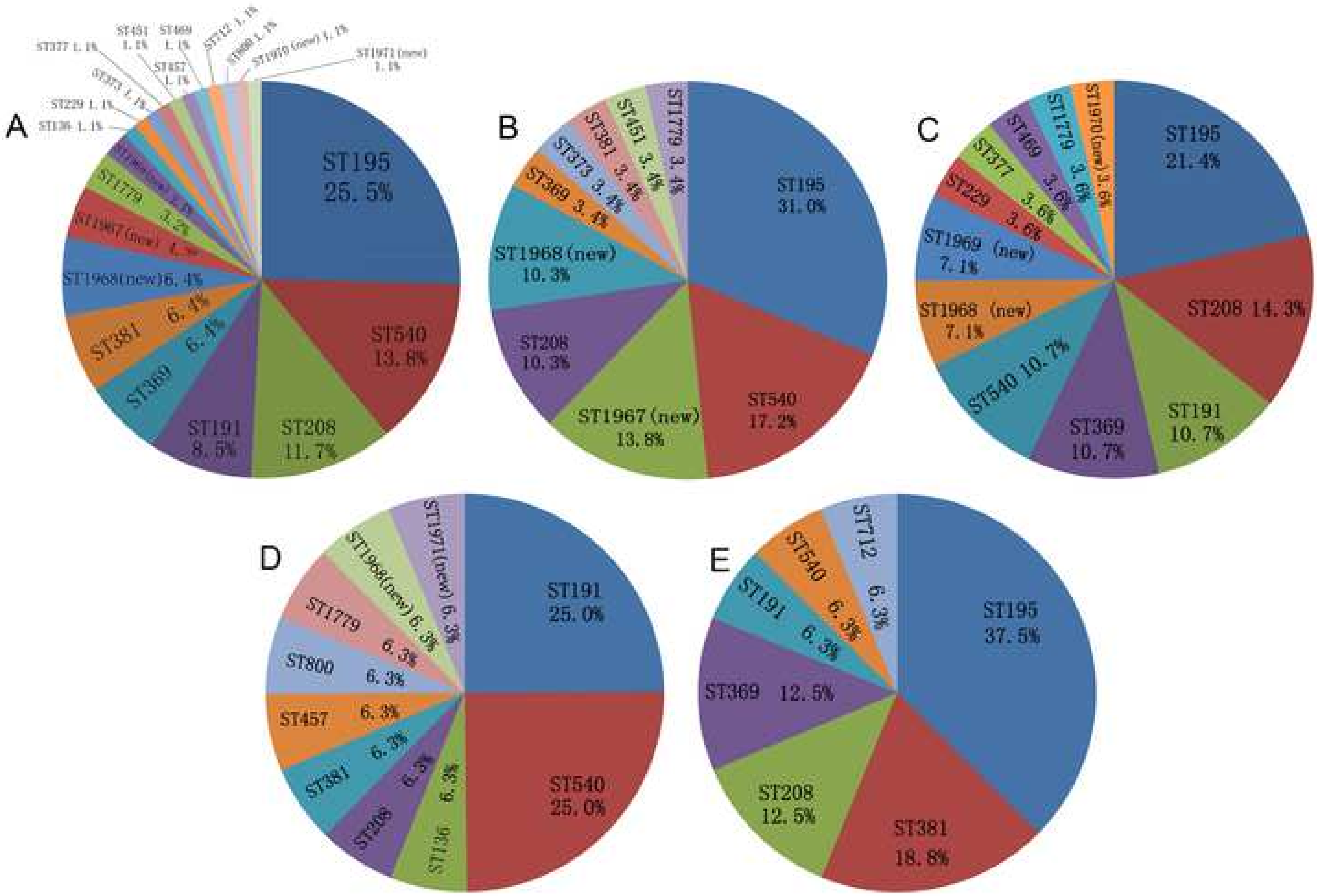

Click here to access/download
Supporting Information
S1 table.docx

Click here to access/download
Supporting Information
S2 table.docx
