# Supplementary material for: Molecular characterization and antibiotic resistance of Acinetobacter baumannii in cerebrospinal fluid and blood
Source: PLoS One. 2021 Feb 22;16(2):e0247418. doi: 10.1371/journal.pone.0247418 (PMC7899338; doi:10.1371/journal.pone.0247418)
Supplement: Attachment — Submitted filename: Response to Reviewers.docx [file pone.0247418.s005.docx]

Dear Editors and Reviewers:

We appreciate the interest that the editors and reviewers have expressed in our manuscript and the constructive comments they have given. Those comments are all valuable and very helpful for revising and improving our paper, as well as the important guiding significance to our researches. We have carefully revised the manuscript according to the insightful comments and provided point-by-point responses as follows, including how and where the text was modified.

**Reviewer #1: Comments to the Author**

The study by Wang et al analysed *A. baumannii* isolates obtained from human CSF and blood samples. The methods used included PCR to detect bla-oxa carbapenemase genes, MLST and AST.

1. The MS needs major language revision.

2. And the figures, especially fig. 3 need to be modified, i.e. the same colour scheme should be applied to the same ST.

3. The MS would benefit enormously if it were shortened to a letter or note or something alike.

4. The methods and analyses used are not new and do not have to be elaborated. Moreover, the results are also not unexpected, i.e. presence of IC II (CC92) in China, presence of highly resistant A.b strains, further clonal expansion.

1. The MS needs major language revision.

**Answer**: We have revised the manuscript and made substantial amendments. Besides, we also submitted the revised manuscript to the language editing service, American Journal Experts to edit the manuscript again. The author professor Kyle Stirling (native speaker) also revised this manuscript. We believe the manuscript has been greatly improved with their helps. Please see the revised manuscript.

2. And the figures, especially fig. 3 need to be modified, i.e. the same colour scheme should be applied to the same ST.

**Answer**: We modified the Fig. 3. The same color schemes were applied to the same ST. We also modified the number of decimals on the Y-axis for Fig. 1. And decimals were changed from two to one which consistent with the results of the manuscript. Please see the revised manuscript and the revised figures.

3. The MS would benefit enormously if it were shortened to a letter or note or something alike.

**Answer**: We appreciate the reviewer’s suggestion. Previous molecular epidemiology studies mainly focus on carbapenem-resisitant *A. baumannii* which were obtained from various sites. Few studies were performed to investigate the molecular characterization and antibiotic resistance of *A. baumannii* in cerebrospinal fluid and blood. As the presence of *A. baumannii* strains in CSF and blood is a critical value and poses great difficulties for clinical treatment options, we think it is worth to describe the molecular characterization and antibiotic resistance in detail for these strains.

In this study, we found that CC92 and *bla*_OXA-23_ gene were predominant among CRAB strains in CSF and blood. Although these results were consistent with other reports from China (CRAB were isolated from various sites). We think these results could represent the molecular characterization for CRAB strains in CSF and blood. Besides, we also observed many new epidemiological data of CSF and blood *A. baumannii* strains. i.e., we found five novel STs. Among them, both of the two ST1969 isolates were positive for the *bla*_OXA-24_ gene a negative for the *bla*_OXA-23_ gene and showed an imipenem-sensitive but meropenem-resistant phenotype. We also found that compared to *A. baumannii* strains isolated from various sites, the resistance rates of CSF and blood *A. baumannii* strains for imipenem and meropenem were much higher. We think these new data may help to improve infection control measures and clinical treatment in hospitals. Therefore, we have simplified the description of Materials and Methods as well as the discussion in the revised manuscript but we still would like to publish the manuscript as the “Research Article” form in the Journal of Plos one.

4. The methods and analyses used are not new and do not have to be elaborated. Moreover, the results are also not unexpected, i.e. presence of IC II (CC92) in China, presence of highly resistant A.b strains, further clonal expansion.

**Answer**: We have simplified the description of Materials and Methods as well as the discussion. Please see the revised manuscript.

**Reviewer #2: Comments to the Author**

This study gives an insight into epidemiology and antibiotic resistance pattern and mechanism of carbapenem of *A. baumannii* strains isolated from CSF and Blood.

1. The study includes 50 isolates from CSF - are all these isolates were true pathogens the contamination has been ruled out to be clarified.

2. Comments are included in the manuscript which can be clarified. 3. Reasons for high resistance level to Carbapenems and low rate of resistance to Tigicycline and levofloxacin to be discussed

4. The manuscript needs correction for English as suggested in the comments in the manuscript.

5. Wheather the treatment and outcome of the patient has been looked into if so which are the drugs used for the treatment of these infections can be mentioned.

1. The study includes 50 isolates from CSF - are all these isolates were true pathogens the contamination has been ruled out to be clarified.

**Answer**: CSF or blood specimens were collected from patients with suspected central nervous system infection or BSIs according to CDC criteria [1]. All these isolates were true pathogens and the contamination has been ruled out. We added this information in the revised manuscript. (Lines 92-94)

2. Comments are included in the manuscript which can be clarified.

**(1) Correct the English**

**Answer**: We corrected this sentence in the revised manuscript. (Lines 79-81)

**(2) Are all the 50 isolates from CSF were confirmed pathogens? and not the contaminants.**

**Answer**: CSF or blood specimens were collected from patients with suspected central nervous system infection or BSIs according to CDC criteria [1]. All these isolates were true pathogens and the contamination has been ruled out. We added this information in the revised manuscript. (Lines 92-94)

**(3) Any increase or decrease in the resistance pattern for carbapenem over the years?**

**Answer**: The changes in resistance rates of imipenem were observed to decrease from 89.7% in 2016 to 89.3% in 2017 and then increased to 93.8% in 2018 and 2019. For meropenem, the resistance rates were observed to increase from 89.7% in 2016 to 96.4% in 2017 and then decreased to 93.8% in 2018 and 2019. We added this information in the revised manuscript. (Lines 136-139)

**(4) needs correction**

**Answer**: We corrected the word “AB73” to “AB13” in the revised manuscript. (Line 211)

3. Reasons for high resistance level to Carbapenems and low rate of resistance to Tigicycline and levofloxacin to be discussed

**Answer**: We calculated the frequency of the usage for carbapenems, tigecycline, colistin and levofloxacin from January 2016 to April 2019. The results showed that the usage frequency for tigecycline and colistin were 10 times and 492 times lower than carbapenems (Table 1). We think this is the reason for high resistance level to carbapenems and low rate of resistance to tigicycline and colistin in this hospital. As levofloxacin also has a high usage frequency, the resistance rate was also upper than tigecycline and colistin. We have added this information in the revised manuscript. (Lines 240-242)

Table 1| The frequency of the usage for carbapenems, tigecycline, colistin and levofloxacin

|  | carbapenems | tigecycline | colistin | levofloxacin |
| --- | --- | --- | --- | --- |
| frequency | 15752 | 1485 | 32 | 54732 |

4. The manuscript needs correction for English as suggested in the comments in the manuscript.

**Answer**: We appreciate the reviewer’s comments and we corrected the English as suggested in the comments in the manuscript. Please see the revised manuscript. (Lines 79-81)

5. Wheather the treatment and outcome of the patient has been looked into if so which are the drugs used for the treatment of these infections can be mentioned.

**Answer**: (1) We looked into the usage of the antibiotics for these 94 patients and calculated the usage rate for 88 patients who infected with CRAB. The results showed that tigecycline has the highest usage rate (more than 70% CRAB patients were treated by tigecycline) which suggested that tigecycline served as a preferred therapeutic agent to control CRAB infections in this hospital. Although colistin also has a low resistance rate, fewer patients were treated by this antibiotic as its negative effect on the patient (Table 2). We added this information in the revised manuscript. (Lines243-246)

Table 2| The usage rate of the antibiotics for patients infected with CRAB

| Antimicrobial | usage frequency | usage rate |
| --- | --- | --- |
| Tigecycline | 62 | 70.50% |
| Cefoperazone/sulbactam | 46 | 52.30% |
| Minocycline | 13 | 14.80% |
| Imipenem | 3 | 3.40% |
| Meropenem | 16 | 18.20% |
| Biapenem | 16 | 18.20% |
| Imipenem/Cilastatin | 6 | 6.80% |
| Mezlocillin/Sulbactam | 4 | 4.50% |
| colistin | 6 | 6.80% |
| Piperacillin/Tazobactam | 5 | 5.70% |
| Amikacin | 20 | 22.70% |
| Etimicin | 10 | 11.40% |
| Gentamicin | 2 | 2.30% |
| Levofloxacin | 15 | 17.00% |
| β-lactams | 11 | 12.50% |

(2) As this is a retrospective study, the follow-up studies were not performed for every discharged patient or patients transferred to another hospital, so we could not evaluated the outcome of these patient. We will perform this study in the future.

References

1.	Garner JS, Jarvis WR, Emori TG, Horan TC, Hughes JM. CDC definitions for nosocomial infections, 1988. American journal of infection control. 1988;16(3):128-40. Epub 1988/06/01. doi: 10.1016/0196-6553(88)90053-3. PubMed PMID: 2841893.
