# Supplementary material for: Molecular characterization and antibiotic resistance of Acinetobacter baumannii in cerebrospinal fluid and blood
Source: PLoS One. 2021 Feb 22;16(2):e0247418. doi: 10.1371/journal.pone.0247418 (PMC7899338; doi:10.1371/journal.pone.0247418)
Supplement: Attachment — Submitted filename: Response to Reviewers.docx [file pone.0247418.s006.docx]

Dear Editors and Reviewers:

We appreciate the interest that the editors and reviewers have expressed in our manuscript and the constructive comments they have given. We have re-scrutinized to improve the English by a language editing service, “LetPub” to edit the manuscript. The figure 1 showed the “Language Editing Certificate”. We also added the information of the antibiotics used for the treatment in S3 table. We believe the manuscript has been greatly improved. Please see the revised manuscript.

**Reviewer #1: Comments to the Author**

**The authors have done a thorough investigation on clinical A. baumannii isolates obtained from CSF and blood at the Shandong Hospital and the results are certainly important in the Chinese and global setting. However, the MS still needs a rigorous language revision to improve readability before it can be considered for publication in PLOS one or other journals.**

**Answer**: Thanks for your comments and suggestion. We have re-scrutinized to improve the English by a language editing service, “LetPub” to edit the manuscript. The figure 1 showed the “Language Editing Certificate”. We believe the manuscript has been greatly improved. Please see the revised manuscript.

**Reviewer #2: Comments to the Author**

**All the reviewer comments are addressed and the manuscript is revised accordingly. The manuscript and the data presented will add therapeutic applicability in the treatment of blood and CSF infections caused by Acinetobacter spp**

**Answer**: We appreciate the reviewer’s comments and suggestion. We added the information of the antibiotics used for the treatment in S3 table and made language revision by using language editing service of “LetPub”. The figure 1 showed the “Language Editing Certificate”. We believe the manuscript has been greatly improved. Please see the revised manuscript.

Fig. 1 Certificate of English Language Editing
